# Modeling the receptor pharmacology, pharmacokinetics, and pharmacodynamics of NKTR-214, a kinetically-controlled interleukin-2 (IL2) receptor agonist for cancer immunotherapy

**DOI:** 10.1371/journal.pone.0179431

**Published:** 2017-07-05

**Authors:** Deborah Charych, Samira Khalili, Vidula Dixit, Peter Kirk, Thomas Chang, John Langowski, Werner Rubas, Stephen K. Doberstein, Michael Eldon, Ute Hoch, Jonathan Zalevsky

**Affiliations:** Nektar Therapeutics, San Francisco, California, United States of America; Stony Brook University, UNITED STATES

## Abstract

Cytokines are potent immune modulating agents but are not ideal medicines in their natural form due to their short half-life and pleiotropic systemic effects. NKTR-214 is a clinical-stage biologic that comprises interleukin-2 (IL2) protein bound by multiple releasable polyethylene glycol (PEG) chains. In this highly PEG-bound form, the IL2 is inactive; therefore, NKTR-214 is a biologic prodrug. When administered *in vivo*, the PEG chains slowly release, creating a cascade of increasingly active IL2 protein conjugates bound by fewer PEG chains. The 1-PEG-IL2 and 2-PEG-IL2 species derived from NKTR-214 are the most active conjugated-IL2 species. Free-IL2 protein is undetectable *in vivo* as it is eliminated faster than formed. The PEG chains on NKTR-214 are located at the region of IL2 that contacts the alpha (α) subunit of the heterotrimeric IL2 receptor complex, IL2Rαβγ, reducing its ability to bind and activate the heterotrimer. The IL2Rαβγ complex is constitutively expressed on regulatory T cells (Tregs). Therefore, without the use of mutations, PEGylation reduces the affinity for IL2Rαβγ to a greater extent than for IL2Rβγ, the receptor complex predominant on CD8 T cells. NKTR-214 treatment *in vivo* favors activation of CD8 T cells over Tregs in the tumor microenvironment to provide anti-tumor efficacy in multiple syngeneic models. Mechanistic modeling based on *in vitro* and *in vivo* kinetic data provides insight into the mechanism of NKTR-214 pharmacology. The model reveals that conjugated-IL2 protein derived from NKTR-214 occupy IL-2Rβγ to a greater extent compared to free-IL2 protein. The model accurately describes the sustained *in vivo* signaling observed after a single dose of NKTR-214 and explains how the properties of NKTR-214 impart a unique kinetically-controlled immunological mechanism of action.

## Introduction

Activation of the immune system is a key component of mounting an effective durable anti-tumor response. The successful clinical use of checkpoint blockade antibodies to overcome immune suppressive mechanisms in the tumor microenvironment has transformed patient care [1–5]. However, most patients do not benefit from antagonist checkpoint blockade, suggesting that certain tumor microenvironments may require the addition of complementary immune agonist mechanisms to overcome tolerance and suppression [6].

Therapeutic agonists based on natural proteins are attractive immune modulators; however, they are not ideal pharmaceutical agents due to poor pharmacokinetics (PK), poor tolerability, and pleiotropic activity that may be exacerbated by frequent dose administration [7, 8]. The cytokine interleukin-2 (IL2) is an endogenous agonist of the IL2 pathway and is a well-described stimulator of CD8^+^ T cell (CD8 T) and NK cells [9]. A high-dose IL2 regimen administered every 8 hours in a hospital setting using an IL2 variant known as ‘aldesleukin’ was approved in the 1990s by the United States Food and Drug Administration for the treatment of metastatic melanoma and renal cell carcinoma, providing up to 25% durable responses [10, 11]. High doses of IL2 are needed to activate CD8 T cells and NK cells, which tend to express the low-affinity IL2 receptor beta gamma subunits (IL2Rβγ). Compounding the need for high doses of IL2 is the poor PK profile of this protein. Despite its legacy as the first approved cancer immunotherapy, high-dose aldesleukin is not broadly used because of severe toxicities associated with over-activation of the immune system. More recent studies indicate that in addition to these toxicities, IL2 also stimulates proliferation and activation of regulatory T cells (Tregs). These cells constitutively express the high-affinity heterotrimeric IL2 receptor alpha beta gamma subunits (IL2Rαβγ). Treg activation may exacerbate immune suppression, potentially compromising the intended anti-tumor response [12–15].

**Fig 1:**
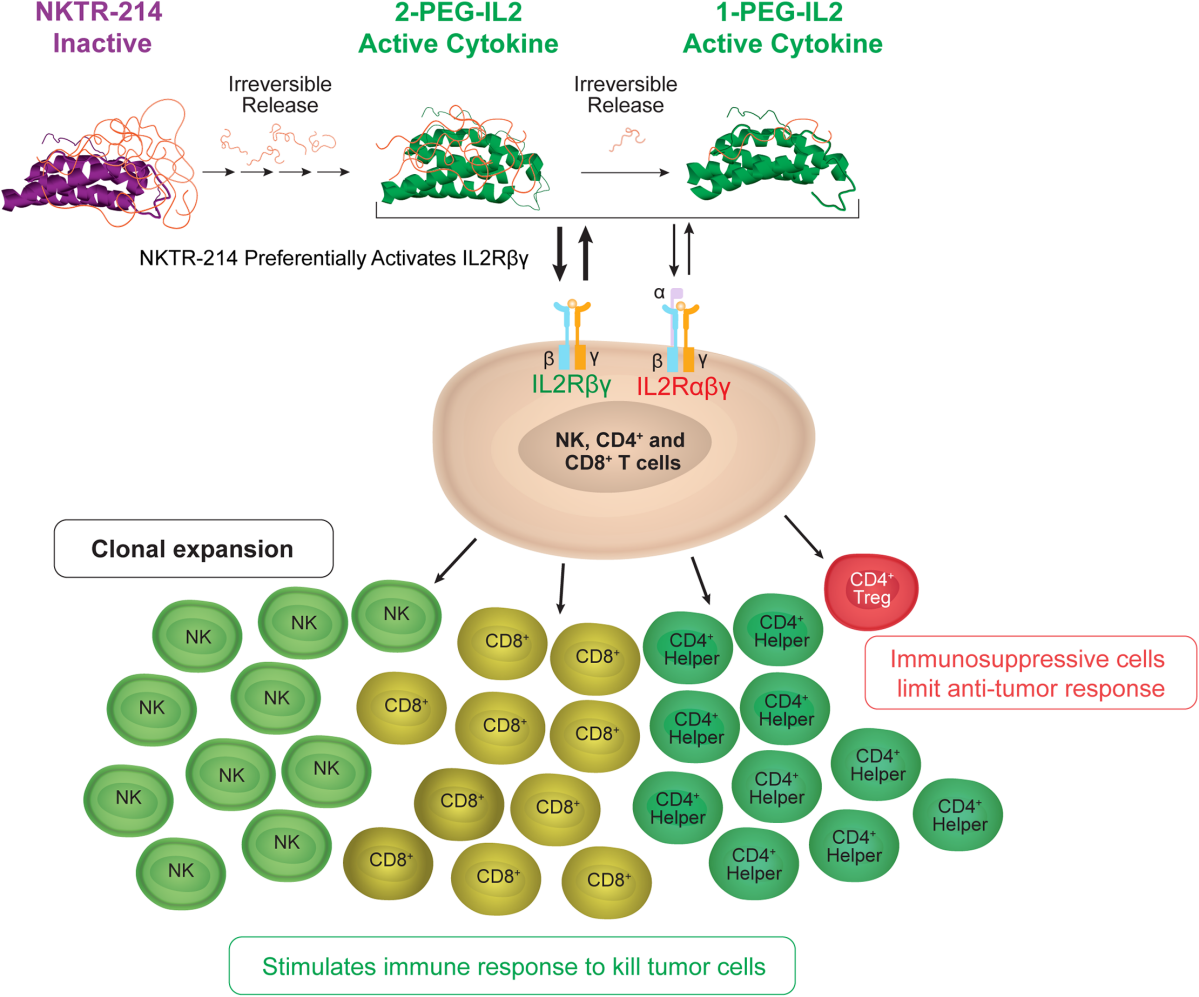
NKTR-214 delivers a controlled, sustained, and biased signal through the IL2 receptor pathway. NKTR-214 is a CD122-biased cytokine agonist conjugated with multiple releasable chains of PEG located at the interface of IL2 and IL2Rαβγ. The PEG chains slowly release at physiological pH, creating conjugated-IL2 species with fewer PEG chains and increased bioactivity. Sustained signaling through the heterodimeric IL2 receptor pathway (IL2Rβγ) preferentially activates and expands effector CD8 T and NK cells over Tregs.

NKTR-214 was invented to harness the potent immune stimulatory benefits of the IL2 pathway to maximize anti-tumor responses and minimize unwanted biological side effects. The design uses the same amino acid sequence of FDA-approved aldesleukin with the addition of multiple releasable polyethylene glycol (PEG) chains located at the region of IL2 that binds to the IL2Rα subunit of the heterotrimeric IL2Rαβγ complex, (Fig 1). When compared to IL2, the differentiating characteristics of NKTR-214 include: 1) receptor bias towards the heterodimeric IL2Rβγ found on CD8 and NK cells leading to markedly higher CD8 to Treg ratios (> 400) in the tumors of murine melanoma; 2) improved single-agent efficacy and synergistic efficacy when combined with checkpoint blockade; 3) improved tolerability with no hypotension or vascular leak syndrome in cynomolgus monkeys at the maximum tolerated dose (MTD); and 4) a PK profile that allows antibody-like dosing regimens [16]. NKTR-214 is currently being evaluated in multiple Phase 1 / 2 clinical trials for the treatment of solid tumors, administered outpatient once every 2 weeks or once every 3 weeks.

This report further defines the mechanism of action of NKTR-214 by mathematically modeling the PK, the biased receptor occupancy, and the sustained downstream intracellular signaling. Anti-tumor efficacy of NKTR-214 in several murine models as a practical outcome of altered pharmacology from novel molecular design is also described. Taken together, the results show how the complex behavior of NKTR-214, a kinetically-controlled biologic prodrug, can be reduced to discrete steps providing a quantitative understanding of its mechanism of action.

## Materials and methods

### Materials

IL2 was cloned, expressed, and purified by Nektar Therapeutics. Unless otherwise stated, “IL2” herein refers to the protein with the FDA-approved amino acid sequence that corresponds to deletion of the N-terminal alanine and single point mutation of cysteine at position 125 to a serine (C125S). This sequence is also referred to by its generic drug name “aldesleukin,” which also has the following synonyms: recombinant interleukin-2 human, rhIL2, and interleukin-2 [17].

NKTR-214 consists of IL2 protein bound by six releasable PEG chains predominantly located at the interface between IL2 protein and its IL2Rα subunit as described previously [16]. Each PEG chain is ~20kDa molecular weight.

Dosing levels throughout this manuscript refer to the IL2 content of NKTR-214. The molecular weight of polyethylene glycol (PEG) is not considered when calculating dosage. Therefore, a dose of 0.8 mg/kg NKTR-214 administered the same amount of IL2 as a dose of 0.8 mg/kg IL2 protein.

### Pharmacokinetic, pharmacodynamic and efficacy *in vivo* studies

PK/PD studies were performed using female C57BL/6 mice (18–20 g) obtained from Charles River Laboratories (Wilmington, MA), n = 5/group per sampling time. Animals received a single intravenous (tail vein) bolus injection of NKTR-214 or aldesleukin. Efficacy studies were performed at Crown Bioscience (Taicang Jiangsu Province, P.R. China), n = 7/group. For the MBT-2 model, female C3H mice (18–21 g; Beijing Vital River Bio-Technology Co. Ltd., China) were implanted in the right flank with 4x10^5^ cells in 0.1 mL of phosphate-buffered saline (PBS) mixed 1:1 with Matrigel®. For the Pan02 model, female C57BL/6 mice (17–19 g; Beijing Vital River Bio-Technology Co. Ltd., China) were implanted in the right flank with 3x10^6^ cells in 0.1 mL of PBS. For the H22 model, female Balb/c mice (18–22 g, Shanghai Lingchang Bio-Technology Co. Ltd., China) were implanted in the right flank with 2x10^6^ cells in 0.1 mL PBS. Animals were administered NKTR-214 by an intravenous (tail vein) bolus injection, 0.8 mg/kg q9dx3. Animal studies were conducted under accreditation by the Association for Assessment and Accreditation of Laboratory Animal Care (AAALAC) and prospectively approved by the Nektar and Crown Bioscience Institutional Animal Care and Use Committees (approval numbers: 2015–004 [PK/PD], AN-1507-009-503 [PanO2], AN-1507-009-481 [H22], and AN-1507-009-470 [MBT-2]).

Each mouse was inoculated with tumor cells subcutaneously at the right lower flank region. Treatment was initiated when the mean tumor size reached 80 – 100mm^3^. After tumor cell inoculation, the animals were checked daily for morbidity, mortality, effects of tumor growth, and effects of treatment on normal behaviors and appearance such as mobility, food and water consumption, eye/hair matting, and any other abnormal effects. Body weight gain / loss was measured twice weekly. Tumor volumes were measured twice weekly in two dimensions using a caliper, and the volume was expressed in mm^3^ using the formula: V = 0.5 *a* x *b*^2^ where *a* and *b* are the long and short diameters of the tumor, respectively. Summary statistics, the mean, and the standard error of the mean (SEM), were calculated for the tumor volumes of each group at every time point. All data are analyzed in GraphPad Prism. All tests are two-sided. P<0.05 is considered to be statistically significant.

Animals were housed in individualized ventilated cages (up to 5 mice per cage) under controlled conditions of temperature, humidity, and light and fed a diet of C0^60^ irradiation sterilized dry granule food. Animals had free access to food during the entire study period. All mice were adapted in the facility for at least 7 days. Euthanasia criteria consisted of either body weight drop of > 20% for 72 hours, hypoactivity, hypothermia, respiratory distress, tumor volume reaching 3000 mm^3^ or termination of the study. At the time of euthanasia, animals were either exsanguinated under isoflurane (terminal time point) with cervical dislocation performed as secondary method to ensure non-recovery or anesthetized by inhalation of 90% CO_2,_ and then euthanized by cervical dislocation.

### Characterization of linker release kinetics

NKTR-214 was brought to pH 7.4 or the indicated pH either by buffer exchange into, or pH adjustment with, 0.5 M sodium phosphate, as indicated. Aliquots were prepared and incubated for varying time periods at the indicated temperatures. Linker release was halted by acidification to pH 4 or below, and samples were held at 4°C until analysis. The extent of linker release was assessed by measuring released free PEG by RP-HPLC. Percentage PEG release or amount of unreleased PEG was determined by referencing a control sample exposed to strong base to achieve 100% release.

### Characterization of binding kinetics and affinity

Binding kinetics and affinity were assessed using surface plasmon resonance (SPR), measured on a Biacore® X-100 instrument (GE Healthcare, Pittsburgh, PA). A CM5 chip (GE Healthcare) was activated using N-hydroxysuccinimide (NHS) and carbodiimide (EDC) per Biacore® protocol, coated with 10 ug/mL anti-human Fc antibody (Thermo Fisher, Waltham, MA) in Hepes buffered saline containing 0.1% Tween 20 (HBSP, GE Healthcare) at a flow rate of 10 uL/min until 5000 Response Units (RU) was immobilized. Next, IL2Rα-Fc or IL2Rβ-Fc or a 1:1 mixture of each pre-incubated for 5–30 minutes (Symansis, Timaru, New Zealand) at 10 ug/mL in HBSP solutions containing 0.1 mg/mL bovine serum albumin (BSA) was immobilized for 3 minutes at 10 uL/min. To measure the kinetic constants, 5 3-fold serial dilutions of the analytes were made starting at 100 nM for IL2 or 300 nM for NKTR-214-AC. These starting concentrations were chosen so that they were at least 10-fold higher than the expected K_d_ values. Analytes were exposed to the receptor-modified chip for exactly 3 minutes each.

The resulting binding curves from the dilution series were fit to a 1:1 model to correlate observed RU to the association and dissociation rate constants, k_a_ and k_d_:

$$R=\frac{k_{a}CRmax}{k_{a}C+k_{d}}\times (1-e^{-(k_{a}C+k_{d})t})$$

The ratio of the dissociation and association rates provides the equilibrium dissociation constant K_d_. Note that in our previous work [16], we used equilibrium binding and an equilibrium binding model to obtain K_d_ for all analytes at the low-affinity IL2Rβ subunit.

### Pharmacokinetics in mice

Mice received a single intravenous injection of NKTR-214 (0.8 mg/kg) or aldesleukin (0.8 mg/kg), n = 5 mice per sampling time. Approximately 200 µL blood was collected in K2EDTA-coated tubes, plasma was separated following centrifugation and frozen at -70°C until analysis. For determination of NKTR-214-RC (all IL2 species derived from NKTR-214, active and inactive), plasma samples were incubated in a pH 9.0 buffer for 16–24 hours at 37°C to hydrolyze the PEG from IL2. The IL2 concentration was then measured using a qualified ELISA. For determination of active conjugated-IL2 derived from NKTR-214 (NKTR-214-AC), an immuno-purification (IP) / strong cation exchange (sCX) extraction process was used. IP used immobilized antibodies specific to IL2 to capture IL2 and PEG-IL2 species from the plasma samples. The captured PEG-IL2 species were bound to sCX resin under conditions that allowed separation of NKTR-214-AC (the most active species derived from NKTR-214 [2-PEG-IL2, 1-PEG-IL2, free-IL2]) from inactive conjugated-IL2 (≥3-PEG-IL2). After extraction, bound PEG was hydrolyzed from IL2 as above and quantified using a qualified ELISA. For determination of 1-PEG-IL2 and potential free-IL2, a similar extraction procedure was used except weak cation resin (wCX) was used to separate the 1-PEG-IL2 from ≥ 2-PEG-IL2 species. Concentration-time profiles were constructed for the relevant conjugated-IL2 species and PK parameters were determined by means of non-compartmental analysis (NCA) using PhoenixWinNonlin 6.4 (Certara USA, Inc., Princeton, NJ). The area under plasma concentration-time curves (AUClast) were calculated using linear-log trapezoidal interpolation and sparse sampling mode and uniform weighting. Terminal half-life (t_½_) was used for extrapolation to infinity. Mean residence time (MRT_inf_) and t_½_ was determined for IL2, NKTR-214-RC, and NKTR-214-AC. For NKTR-214-AC, maximum plasma concentration (C_max_) and time to peak (T_max_) was also determined.

### Phosphorylated signal transducer and activator of transcription 5 (pSTAT5) pharmacodynamics and flow cytometry in mice

Mice received a single intravenous injection of NKTR-214 or aldesleukin, 0.8 mg/kg, n = 5 mice per sampling time point. Blood (100 uL) was treated with 20 volumes (2.0 mL) of pre-warmed Lyse/Fix Buffer (BD Phosflow™, cat # 558049) and incubated for 10 min at 37^°^C. Cells were spun for 8 minutes at 500 g and washed twice with 1.0 mL flow cytometry buffer (eBioscience, Cat # 00-4222-26). After the second wash, cells were permeabilized on ice for 20 minutes with 1.0 mL chilled 90% methanol. Cells were washed once and blocked (Purified Anti-Mouse CD16/CD32 Mouse BD Fc Block™, Cat # 553142) on ice for 30 minutes, followed by staining with Hamster anti-mouse CD3e-PE, clone 500A2 (BD Bioscience Cat # 553240) and Anti-Stat5 (pY694)–Alexa647 (BD Bioscience Cat # 562076) at 4^°^C overnight. Cells were washed, and data acquired on Accuri™ (BD Biosciences) and analyzed using FlowJo® software (FlowJo, LLC).

### Mechanistic model methods

**Fig 2:**
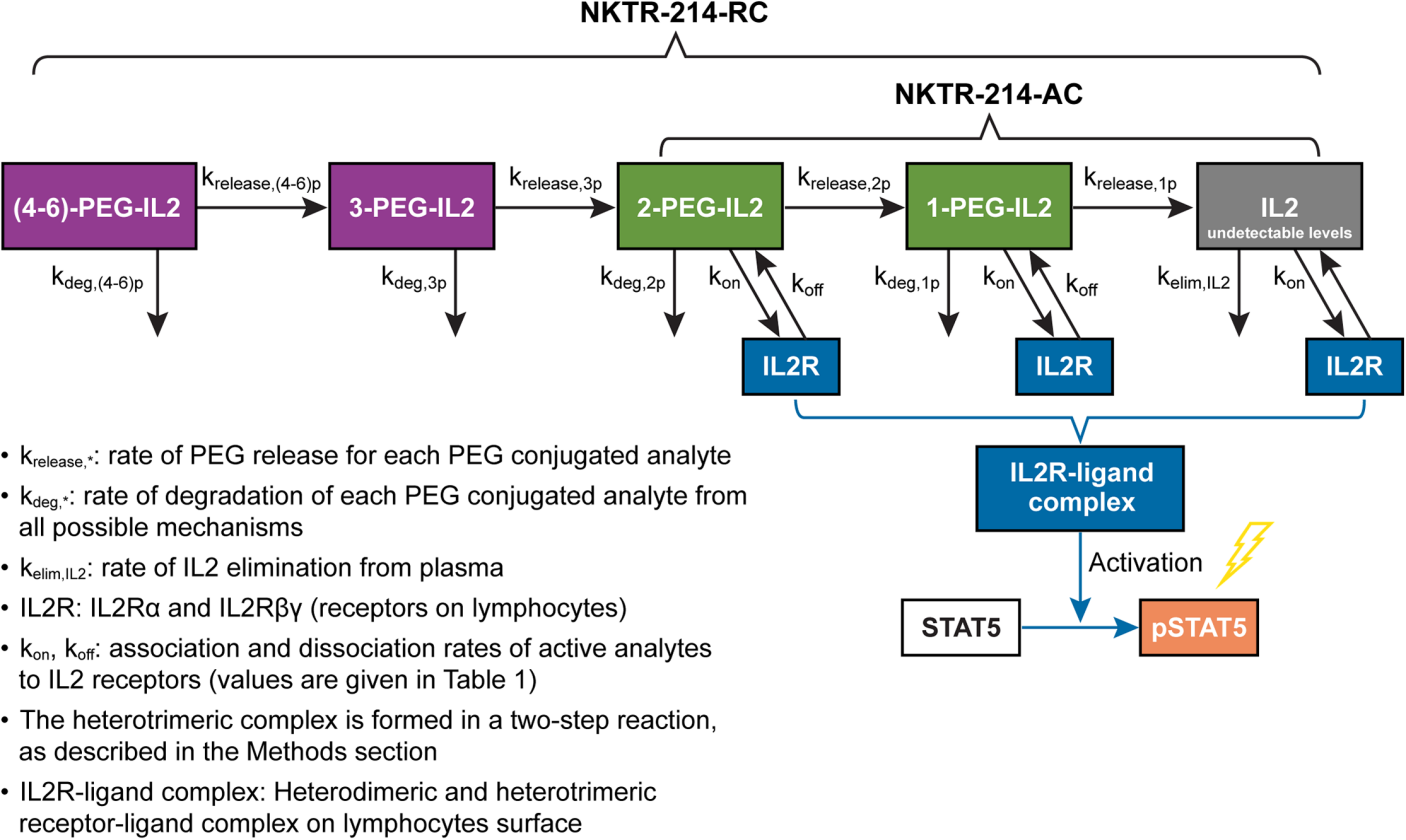
Parameterization of the mathematical model for NKTR-214 dynamics to simulate concentration-time profiles of conjugated-IL2 species derived from NKTR-214 and to describe receptor occupancy of the conjugated-IL2 species at the IL2Rαβγ and IL2Rβγ.

The mechanistic model was developed in Matlab® (Mathworks, MA), using ordinary differential equations (ODE) solvers such as ODE15s, genetic algorithms (GA), and nonlinear solvers such as “fmincon” for optimization. ODE were used to mathematically describe the dynamics of NKTR-214 kinetic processes, IL2 receptor occupancy, and pSTAT5 signaling. The mathematical model incorporated rates for release of PEG chains (k_release_) plus other combined mechanisms (clearance, tissue distribution, etc.) for disappearance from plasma (K_deg_), and binding (k_on_ and k_off_) to the IL2 receptors (Fig 2). The following set of ODEs captured the dynamics of 1-PEG-IL2. Similar sets of equations capture the dynamics of all analytes in the mathematical model:

$$\begin{array}{l} \frac{d[1PEGIL2]}{dt}=k_{release,2peg}∙[2PEGIL2]-k_{release,1peg}∙[1PEGIL2]-k_{elimination}∙[1PEGIL2]\\ \;-k_{on,1peg,\alpha }∙[1PEGIL2]∙[IL2R\alpha ]+k_{off,1peg,\alpha }∙[1PEGIL2.IL2R\alpha ]\\ \;-k_{on,1peg,\beta }∙[1PEGIL2]∙[IL2R\beta ]+k_{off,1peg,\beta }∙[1PEGIL2.IL2R\beta ] \end{array}$$

$$\frac{d[IL2R\alpha ]}{dt}=-k_{on,1peg,\alpha }∙[1PEGIL2]∙[IL2R\alpha ]+k_{off,1peg,\alpha }∙[1PEGIL2.IL2R\alpha ]$$

$$\begin{array}{l} \frac{d[IL2R\beta ]}{dt}=-k_{on,1peg,\beta }∙[1PEGIL2]∙[IL2R\beta ]+k_{off,1peg,\beta }∙[1PEGIL2.IL2R\beta ]\\ \;-k_{on,1peg,\alpha \beta }∙[1PEGIL2.IL2R\alpha ]∙[IL2R\beta ]+k_{off,1peg,\alpha \beta }∙[1PEGIL2.IL2R\alpha \beta ] \end{array}$$

$$\frac{d[1PEGIL2.IL2R\alpha ]}{dt}=k_{on,1peg,\alpha }∙[1PEGIL2]∙[IL2R\alpha ]-k_{off,1peg,\alpha }∙[1PEGIL2.IL2R\alpha ]$$

$$\frac{d[1PEGIL2.IL2R\beta ]}{dt}=k_{on,1peg,\beta }∙[1PEGIL2]∙[IL2R\beta ]-k_{off,1peg,\beta }∙[1PEGIL2.IL2R\beta ]$$

$$\frac{d[1PEGIL2.IL2R\alpha \beta ]}{dt}=k_{on,1peg,\alpha \beta }∙[1PEGIL2.IL2R\alpha ]∙[IL2R\beta ]-k_{off,1peg,\alpha \beta }∙[1PEGIL2.IL2R\alpha \beta ]$$

Model fitting and optimization was based on the PK data as described in the Results. The following statements were assumed to be true in the development of the model:

NKTR-214 is inactive when all 6 PEG chains are attached. Consistent with previously described data [16], the active conjugated-IL2 species derived from NKTR-214 (≤ 2-PEG-IL2) bind and engage the IL2 receptors. To simplify the mathematical model, conjugated-IL2 species with 4, 5 and 6 PEG chains were combined into one species: (4–6)-PEG-IL2.

1. PEG release from NKTR-214 occurs sequentially, and *in vivo* release rates are within 20% of the release rates determined in buffer at pH 7.4. To determine the *in vitro* release rates, data previously described [16] were fit to an ODE model, which assumed first-order kinetics for PEG release. The ODE model was developed in Matlab™ and incorporated individual release rates for each step of the PEG release cascade in contrast to the previously reported fixed rate for PEG release. Using this model, the release rates were estimated by the least squares method as follows: 0.044 hr^-1^, 0.035 hr^-1^, 0.058 hr^-1^, and 0.040 hr^-1^ for release of the initial 1–3 PEG combined, and each additional PEG thereafter.
2. A simplified 1-compartment model is sufficient for modeling plasma PK for NKTR-214. Although prevoius studies have demonstrated substantial tumor exposures of NKTR-214 [16], for the purposes of developing the initial mechanistic dynamics, we constrained the plasma PK profile to a 1-compartment model.
3. The concentration-time profiles for the total and active conjugated-IL2 species derived from NKTR-214 (NKTR-214-RC and NKTR-214-AC) can be constructed from the model-calculated concentration-time profiles of the individual species derived from NKTR-214. Therefore, a) the sum of (4–6)-PEG-IL2, 3-PEG-IL2, 2-PEG-IL2, 1-PEG-IL2, and IL2 fits to the NKTR-214-RC data (all species derived from NKTR-214), b) the sum of 2-PEG-IL2, 1-PEG-IL2, and IL2 fits to the NKTR-214-AC data, and c) the sum of 1-PEG-IL2 and IL2 fits to the 1-PEG-IL2 data. IL2 released from NKTR-214 follows the expected concentration-time profile of aldesleukin.
4. IL2 receptor binding occurs on the surface of lymphocytes between active species derived from NKTR-214 and IL2 receptors based on the SPR determined k_on_ and k_off_ rates. The average concentration of lymphocytes in mice is 3.9 cells/nL. The average number of IL2Rα subunits is 10000 and the average number of IL2β is 1000 [18–21] per lymphocyte.
5. The gamma chain of the IL2R complex contributes to the overall binding affinity of IL2 at the receptor complex. IL2Rγ increases the affinity of IL2 to IL2Rβ by 100-fold (to 1nM) and to IL2Rαβ by 10-fold (to 10 pM) [22, 23]. While the SPR measurements are quantitative, they do not capture the dynamic conformational shifts of the cell surface receptors where all 3 receptor subunits are expressed. The model therefore provides a constant 10-fold decreased K_d_ (increased affinity) for each analyte as measured (IL2, 1-PEG-IL2, and 2-PEG-IL2), at each receptor to account for the gamma-chain receptor
6. The dynamics of receptor binding are previously described for IL2 [13, 20, 24, 25]. Briefly, the heterotrimeric receptor, IL2Rαβγ, is engaged after IL2, 1-PEG-IL2, or 2-PEG-IL2 bind to the alpha subunit, with subsequent recruitment of the beta-gamma complex to form IL2Rαβγ.
7. Occupancy of IL2Rβγ or ILRαβγ is required for phosphorylation and activation of STAT5 [13]. CD3+ T cells account for 40% of total lymphocytes in mice. Experimentally, pSTAT5 was enumerated on CD3+ T cells. To convert receptor concentrations on lymphocytes to CD3+ T cells, the simulated receptor occupancies were multiplied by 0.4. An indirect response model was used to relate the time profile of receptor occupancy to the measured value of pSTAT5 over the time course.

## Results

### *In vitro* release of PEG chains from NKTR-214 follows predictable kinetics

**Fig 3:**
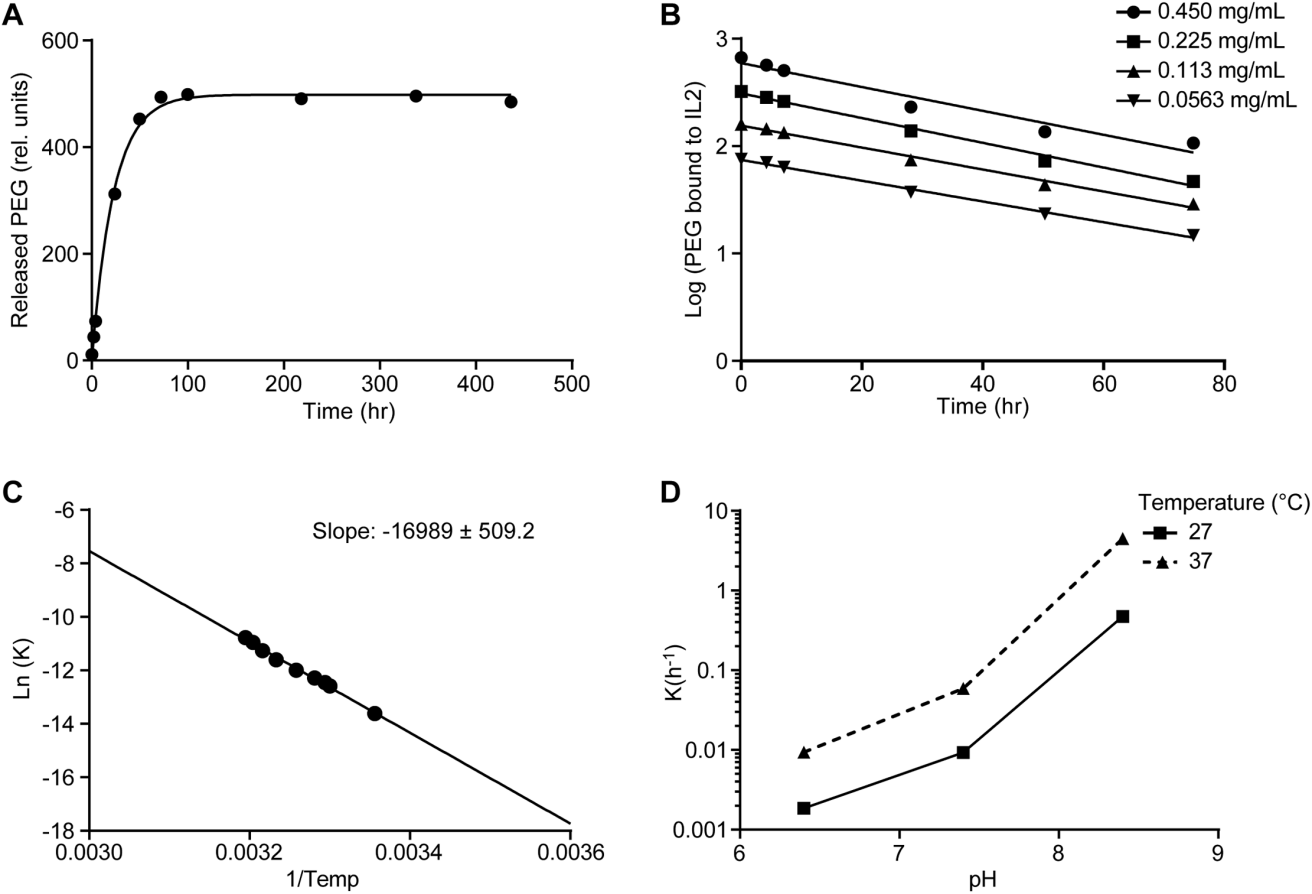
Release of PEG chains follows first-order kinetics. A. Free PEG detected at various time points during incubation in 0.5 M sodium phosphate buffer at pH 7.4 and 37°C (circles) fit to first-order kinetic profile (solid line), B. Semi-log plot of non-released PEG in phosphate buffer at pH 7.4 starting from indicated concentrations of NKTR-214 at pH 7.4 and 37°C. C. Arrhenius plot of Ln(K) versus 1/Temp is linear. Calculated E_a_ = 130 kJ/mole for PEG release, D. Effect of pH and temperature on PEG release rate–an equal volume of 0.5 M sodium phosphate buffer at predetermined pH was added to conjugate to produce the final pH of 6.4 or 7.4.

NKTR-214 is inactive when all 6 PEG chains are attached and does not bind to any IL2 receptors. NKTR-214 was designed to be stable at pH < 6 and to slowly release PEG chains at physiological pH, providing a gradual increase of bioactivity [16]. A quantitative method was developed and implemented to study the PEG release under a variety of controlled conditions. In the method described here, released PEG chains (no longer bound to IL2) were quantified over time using HPLC-UV. Non-linear regression assuming first-order kinetics gave a good fit to the observed complete time course of PEG chain release (Fig 3A) and a rate constant of 0.044 h^-1^ at pH 7.4 and 37°C.

To further confirm the first-order kinetics of PEG chain release, the PEG-release time course was studied using variable initial concentrations of NKTR-214. Similar slopes on a semi-log plot were observed, confirming that the rate constant is independent of starting concentration (Fig 3B), and consistent with first-order kinetics. The temperature dependence of the rate constant was determined by measuring the PEG chain release rate constant at pH 7.4 using a range of temperatures from 25°C to 40°C. The rate constant followed the Arrhenius law, with calculated activation energy (E_a_) for PEG chain release of 130 kJ/mole (Fig 3C). At 37°C, PEG chain release occurred more rapidly at physiological pH 7.4 compared to pH 6.4 (Fig 3D) with rate constants at of 0.059h^-1^ and 0.009h^-1^ and t_½_ of 12 hours and 77 hours, respectively.

### Characterization of receptor interactions

**Fig 4:**
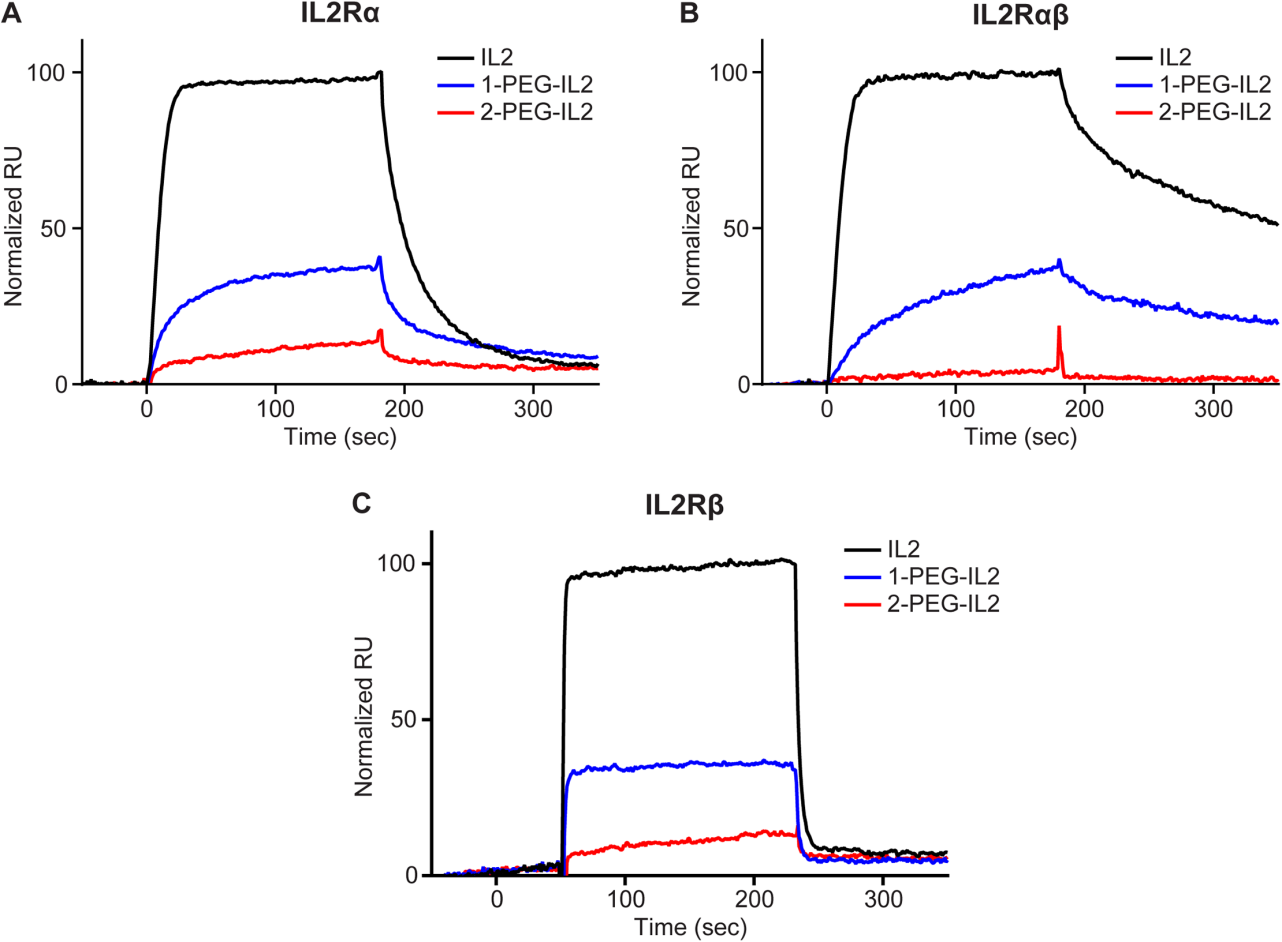
The effect of PEGylation is greatest at the alpha-containing IL2 receptors. Overlay of RU signals normalized by percentage of IL2 response. Each overlay depicts relative response of 1-PEG-IL2 and 2-PEG-IL2 at A. IL2Rα, B. IL2Rαβ, and C. IL2Rβ. Representative data are shown for each analyte at a concentration that is near the respective IL2 K_d_ concentration per Table 1.

Our previous work confirmed the prodrug design of NKTR-214. NKTR-214 was unable to bind or activate any IL2 receptor component as determined by SPR and cellular signaling of murine CTLL2 cells [16]. Conjugated-IL2 bound by ≥ 3-PEG chains was likewise inactive. The most active conjugated-IL2 species derived from NKTR-214 were 1-PEG-IL2 and 2-PEG-IL2, which are formed towards the end of the PEG release cascade. To more fully understand the kinetic differences between binding at IL2Rαβγ and IL2Rβγ receptors, the association and dissociation rates of free-IL2, 1-PEG-IL2, and 2-PEG-IL2 were compared in detail using a modified and optimized SPR methodology (Table 1). In this methodology, receptor subunits are bound to the chip surface to model the cell surface receptors in a simplified controlled system. Given that it was not possible to add the third receptor subunit IL2Rγ to the chip without losing signal intensity [26, 27] we used a 1:1 mixture of IL2Rα and IL2Rβ to mimic cellular IL2Rαβγ whereas IL2Rβ alone at high density was used on the chip to mimic cellular IL-Rβγ. When bound to IL2Rα on cell surfaces, IL2 undergoes a conformational change that allows high-affinity recruitment of the IL2Rβγ subunits [25, 28]. Therefore, we also characterized the binding at IL2Rα alone. The affinity of IL2 at IL2Rα, IL2Rβ and a mixture of IL2Rα and IL2Rβ was similar to previously reported values in the literature [23, 27, 29]. The results for 1-PEG-IL2 and 2-PEG-IL2 were quite different. The degree of PEG conjugation impacted the association rate (k_on_) such that the presence of the PEG chain resulted in significantly slower on-rate for IL2Rαβ. In contrast, the association rate was significantly less affected by PEG conjugation for the IL2Rβ, consistent with the distal site of PEG conjugation at the interaction site between IL2 and IL2Rα. The dissociation rate (k_off_) is relatively less affected by the presence of PEG. Accordingly, compared to IL2, the equilibrium dissociation constant K_d_ for 1-PEG-IL2 is increased by 7.4-fold at IL2Rβ and ~3200-fold at IL2Rαβ. The effect can be further visualized by the representative response plots shown in Fig 4. The association and dissociation kinetic profile of IL2 and active conjugated-IL2 species derived from NKTR-214 are shown at a single concentration near the K_d_ value of IL2 at the indicated receptor (normalized to an IL2 response of 100%). At the alpha-containing receptors, the shallower slope in the association phase and the sharper drop-off in the dissociation is indicative of PEG slowing the association rate and hastening the dissociation rate relative to IL2. In contrast, at IL2Rβ the association rate is less affected by PEG as the slope is similar for all analytes. Note that the concentration of analytes used for illustrating these raw kinetic profiles are not necessarily those used for optimally deriving the values of K_d_ shown in Table 1.

**Table 1:** The effect of PEGylation is greatest at the IL2Rαβ complex bound to the SPR chip. Summary of binding kinetics for IL2 and active conjugated-IL2 species derived from NKTR-214 at the human IL2Rα, IL2Rβ, or IL2Rαβ. IL2Rαβ simulates the heterotrimeric IL2Rαβγ, whereas IL2Rβ simulates the heterodimeric IL2Rβγ.

Receptor	Analyte	k_on_ (M^-1^sec^-1^)	k_off_ (sec^-1^)	K_d_ (nM)
	IL2	4.84x10^7^	0.415	8.57
**IL2Rα**	1-PEG-IL2	8.33x10^5^	0.158	189.7
** **	2-PEG-IL2	3.74x10^5^	0.182	486.6
** **	IL2	1.46x10^8^	0.014	0.096
**IL2Rαβ**	1-PEG-IL2	3.56x10^5^	0.111	311.8
** **	2-PEG-IL2	2.41x10^5^	0.108	448.1
** **	IL2	1.26x10^6^	0.301	238.9
**IL2Rβ**	1-PEG-IL2	2.82x10^5^	0.499	1769.5
	2-PEG-IL2	1.66x10^5^	0.656	3951.8

### Pharmacokinetics of NKTR-214

**Fig 5:**
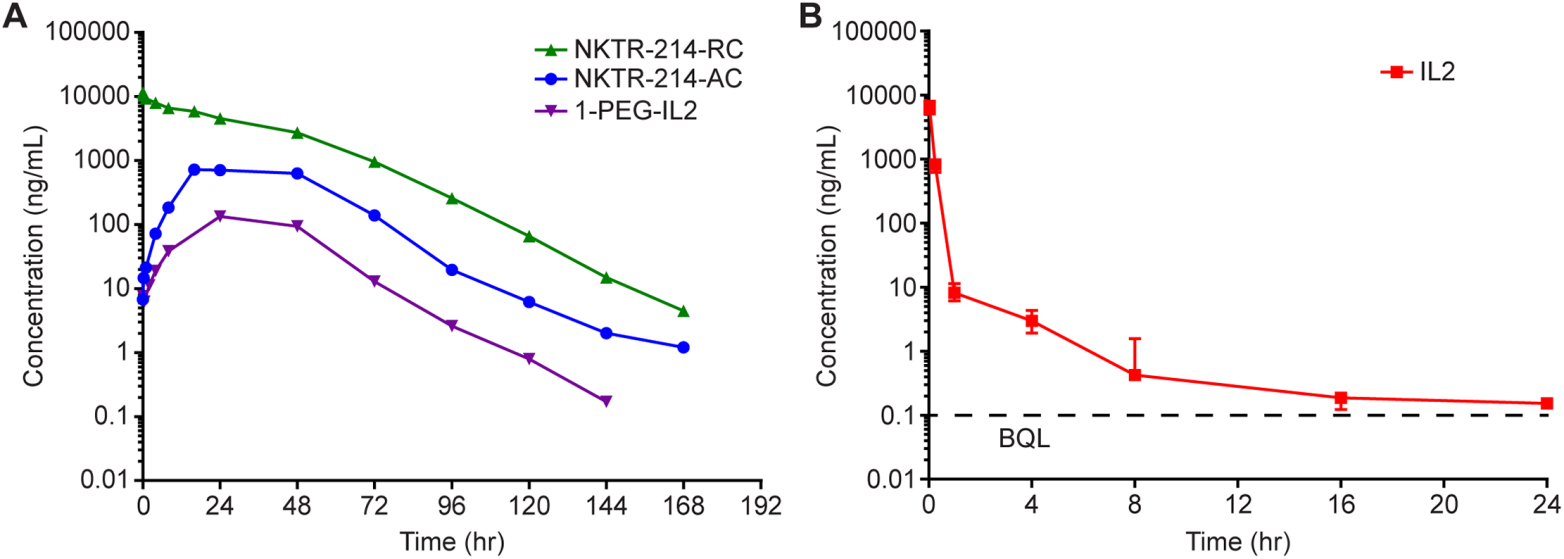
A single dose of NKTR-214 leads to sustained exposure. A. Semi-logarithmic plot of plasma concentration vs. time curves for NKTR-214-RC, NKTR-214-AC, and 1-PEG-IL2 after administration of NKTR-214 B. Semi-logarithmic plot of plasma concentration of IL2 after administration of aldesleukin.

At physiological pH, NKTR-214 evolves over time into an array of conjugated-IL2 species derived from the starting prodrug NKTR-214 (Fig 1). The effect is fundamentally different than a slow release of free-IL2 from a formulated matrix [30, 31] or from free-IL2 linked to an Fc to improve t_½_ [32, 33]. Therefore, we developed specialized bioanalytical assays to characterize relevant groups of conjugated-IL2 species derived from NKTR-214. One assay for NKTR-214-RC captures all IL2-containing conjugates independent of the number of PEG chains attached, encompassing active and inactive species. A second assay for NKTR-214-AC focuses on quantification of the most active species derived from NKTR-214 (ie, 2-PEG-IL2, 1-PEG-IL2, and free-IL2). A third assay quantifies only 1-PEG-IL2 and free-IL2, and a fourth quantifies only free-IL2. Although free-IL2 is part of the assays, it is not detectable in plasma following administration of NKTR-214 due to its slow formation rate relative to the fast clearance of IL2. After intravenous administration of NKTR-214 in mice, NKTR-214-RC declined bi-exponentially with a t_½_ of 15.5 hours (Fig 5A and Table 2). Consistent with the *in vitro* release kinetics described above, the active species (NKTR-214-AC) gradually increased, reaching C_max_ 16 hours post dose, and decrease with a t_½_ of 17.6 hours. As expected from step-wise release of PEG chains, 1-PEG-IL2 was the last conjugated-IL2 form to reach its C_max_, 24 hours after administration of NKTR-214. The 1-PEG-IL2 concentration declined thereafter with a t_½_ of 11 hours. Pharmacokinetics of NKTR-214 and conjugated-IL2 species derived from NKTR-214 are in stark contrast to those of aldesleukin administration (Fig 5B), which is eliminated with a t_½_ of 4 hours. Compared to aldesleukin, the most active conjugated-IL2 species derived from NKTR-214 (ie, 2-PEG-IL2 and 1-PEG-IL2) together have a 10-fold lower C_max_, while AUC was 27-fold higher, t_½_ was 4-fold longer, and mean residence time (MRT) was 190-fold longer (Table 2).

**Table 2:** Mean (±SE) plasma PK parameters after administration of NKTR-214 or aldesleukin, each at 0.8 mg/kg, i.v.

	Aldesleukin 0.8 mg/kg IV	NKTR-214 0.8 mg/kg, IV			Ratio ^1^
	IL2	NKTR-214-RC	NKTR-214-AC	1-PEG-IL2	NKTR-214-AC / IL2
**C**_**max**_ **(μg/mL)**	6.6 ± 0.51	11.6 ± 0.78	0.72 ± 0.06	0.13	0.11
**T**_**max**_ **(hr)**	NA	NA	16	24	
**AUClast** ^**1**^ **(μg.hr/mL)**	1.38 ± 0.07	308 ± 7.18	37.8 ± 1.84	5.81	27
**t**_**½**_**(hr)**	4.0	15.5	17.6	10.8	4.4
**MRTinf (hr)**	0.20	28.0	37.6	35.7	190

^1^The ratio provides a measure of the indicated PK parameter of active conjugated-IL2 derived from NKTR-214 compared to the PK parameter from an equivalent dose of IL2 (aldesleukin)

Due to sparse sampling, SE was calculated for only C_max_ and AUClast; NA: not applicable

### Sustained and controlled pSTAT5 activation from NKTR-214 *in vivo*

**Fig 6:**
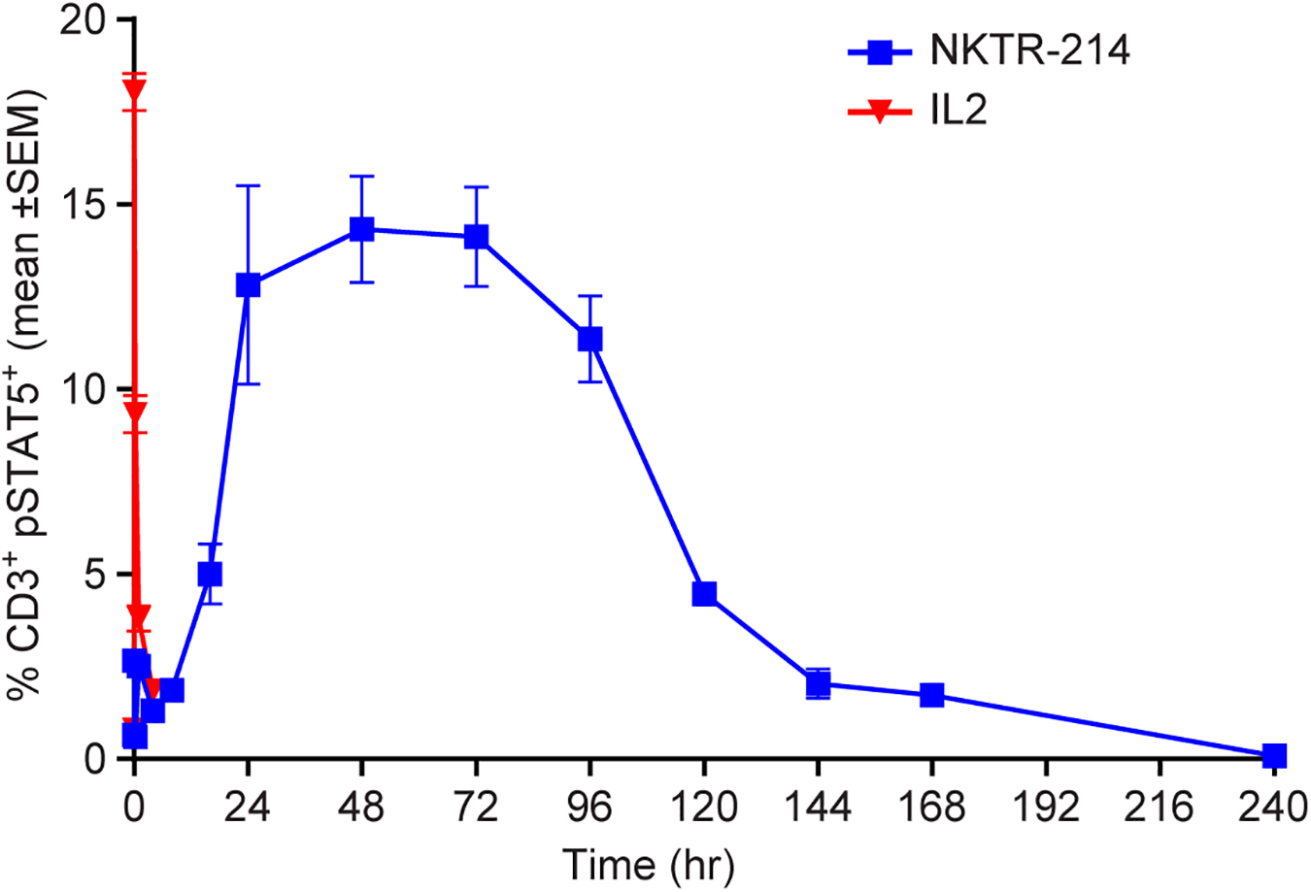
NKTR-214 delivers a controlled and sustained activation signal to the IL2 pathway *in vivo* measured by pSTAT5 levels in whole peripheral blood. Mice were treated with a single 0.8 mg/kg dose of either aldesleukin (red) or NKTR-214 (blue) and monitored for up to 240 hours. Peripheral blood was collected at the indicated time points post-dose and pSTAT5 in lymphocyte populations was determined by flow cytometry using antibodies to intracellular pSTAT5 and to extracellular surface marker CD3. N = 5 mice per time point per group.

To study the PD effects of IL2 pathway activation, we monitored the time, duration, and magnitude of phosphorylation of signal transducer and activator of transcription 5 (STAT5) in peripheral blood T cells following a single intravenous administration of NKTR-214 or aldesleukin in naïve mice. A single dose of aldesleukin produced a rapid and robust increase in phosphorylated STAT5 (pSTAT5), detectable within 2 minutes after dosing and resolving to baseline within 4 hours. In contrast, a single dose of NKTR-214 resulted in a slower rise of pSTAT5 levels, detectable above baseline ~24 hours post dose. The signal reached maximal effect by 48 hours and remained elevated for up to 7 days (Fig 6). The time-course of pSTAT5 activation after treatment with aldesleukin and NKTR-214 was closely aligned with their respective PK profiles. While the maximum percentage of activated pSTAT5+ CD3+ cells was similar after either aldesleukin or NKTR-214 (approximately 15%), the duration of activation with NKTR-214 was ~13-fold greater compared to aldesleukin as measured by total area under the effect curve (1399 ± 70.9%-hrs vs. 108 ± 8.7%-hrs for NKTR-214 and aldesleukin, respectively).

### Mechanistic model for NKTR-214 *in vivo* dynamics

NKTR-214 has 3 prominent mechanistic features: 1) biased receptor binding such that PEGylation impacts binding at IL-2Rαβγ to a greater extent than IL-2Rβγ relative to IL2; 2) sustained release of an array of conjugated-IL2 species; and 3) sustained activation of the IL2 pathway. While these effects were measured and reported separately in the previous sections, a mathematical model was developed to unify the data to: 1) describe the concentration-time profiles for NKTR-214-RC, NKTR-214-AC, and the individual conjugated-IL2 species that comprise these subsets; 2) quantify the receptor bias by calculating the anticipated *in vivo* receptor occupancy at each IL2 receptor complex; and 3) relate the output of the mechanistic model to the downstream PD response. The model provides a quantitative approach to describe the kinetically-controlled dynamics of IL-2 receptor agonism by NKTR-214.

NKTR-214 has 6 PEG chains attached to IL2 and can liberate conjugated-IL2 species where the IL2 protein is bound by 5-PEGs, 4-PEGs, 3-PEGs, 2-PEGs, or 1-PEG. Theoretically, free-IL2 can also be formed even though it is not detectable *in vivo*. Conjugated-IL2 species with 6, 5, and 4 PEG chains were not independently considered in the model because they are not active and do not bind or activate downstream signaling [16]. These have been grouped as one species defined as (4-6-PEG-IL2). The mathematical model was based on the reaction cascade shown in Fig 2, incorporating PEG release rates, degradation rates for each conjugated species, and receptor binding kinetics (k_on_ and K_off_), as well as IL2 elimination and distribution rates. The values for unknown parameters were constrained based on the assumptions listed in the Methods section.

### Model fit to PK data and simulation of concentration-time profiles

**Fig 7:**
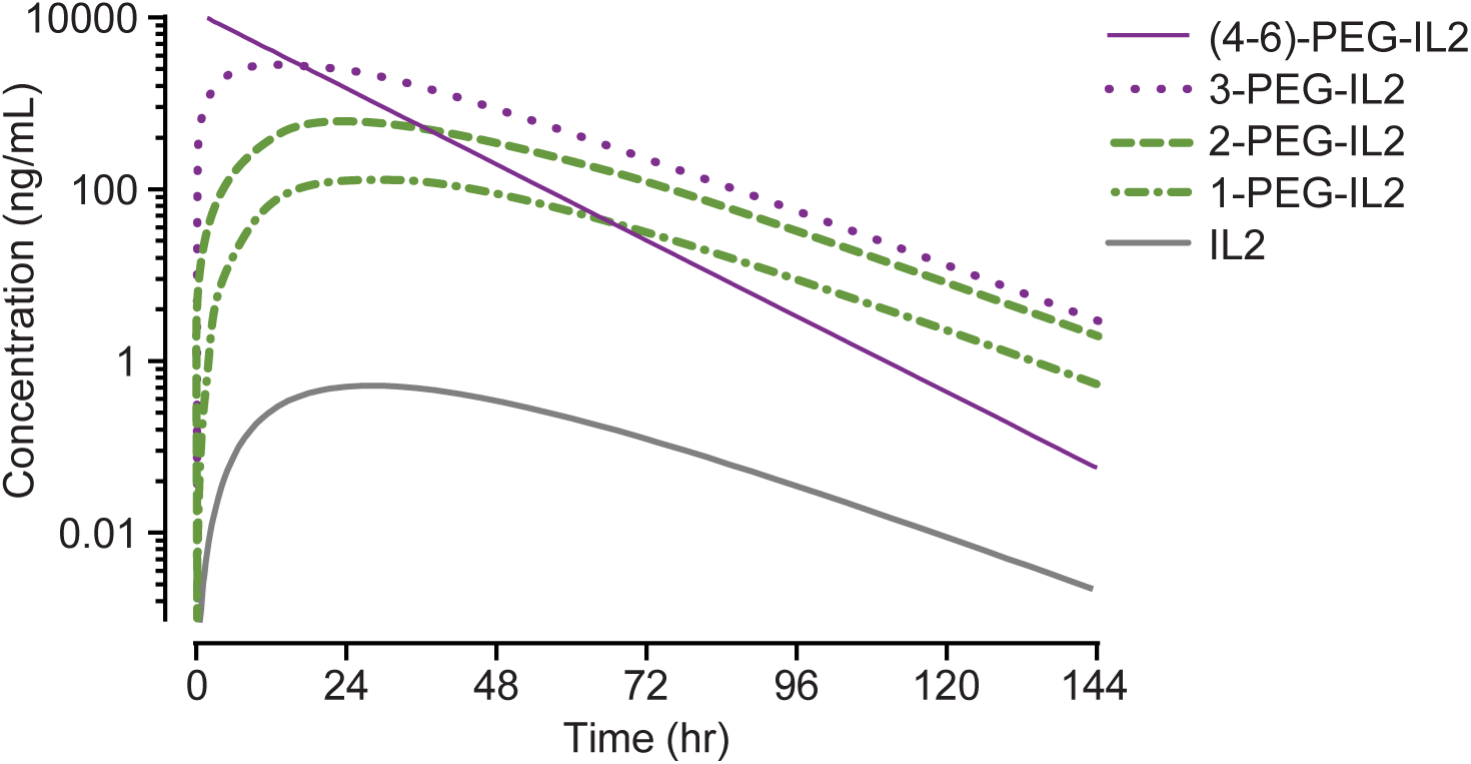
Model-calculated concentration–time profile for species of conjugated-IL2 or free-IL2 that could be theoretically generated from a single dose of 0.8 mg/kg NKTR-214. Where data are available, the model agrees closely with the PK data shown in Table 3. The model calculates that administration of NKTR-214 results in sustained concentrations of conjugated-IL2 with exceedingly low concentrations of free-IL2, consistent with its fast *in vivo* clearance and slow formation from NKTR-214.

**Fig 8:**
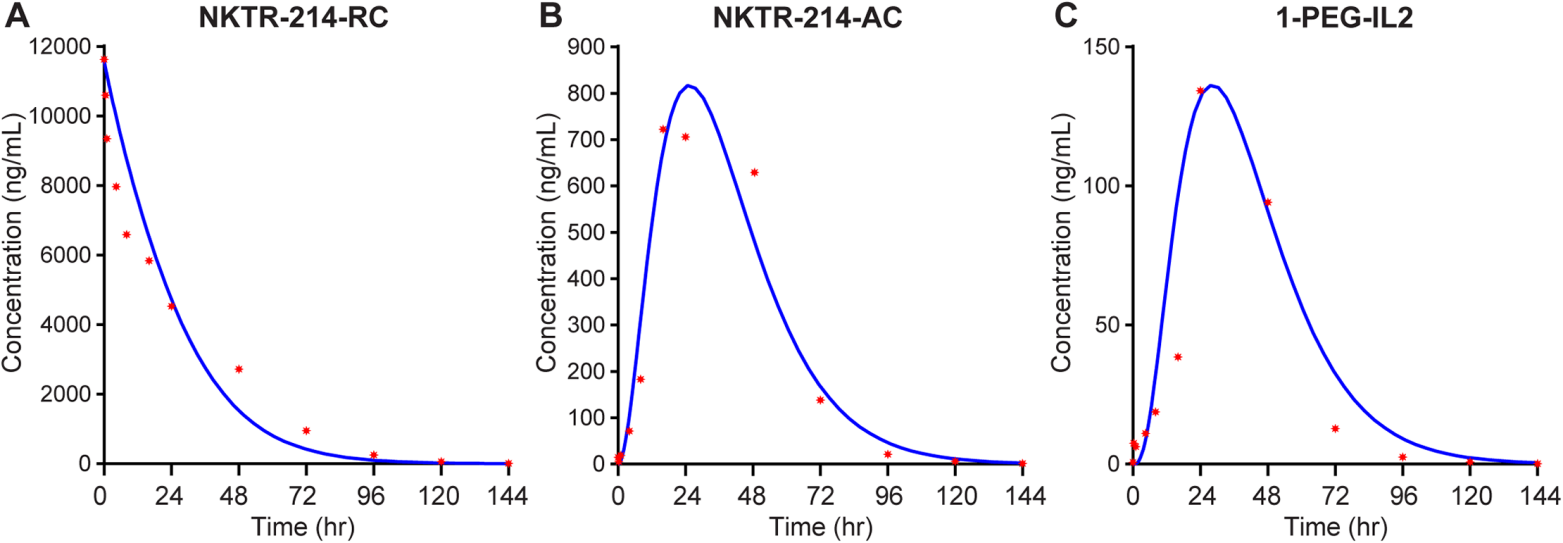
The model suitably fits to measured NKTR-214 concentration-time profiles. Summing the concentration-time profile of the individual model-derived conjugated-IL2 fit well to the experimentally determined concentration-time profiles of A. NKTR-214-RC, B. NKTR-214-AC, and C. 1-PEG-IL2, the most active conjugate derived from NKTR-214. Solid lines represent the simulation and individual red symbols represent the experimental data.

Fig 7 shows the concentration-time profiles for (4–6)-PEG-IL2, 3-PEG-IL2, 2-PEG-IL2, 1-PEG-IL2, and free-IL2 as simulated by the model. The calculated PK parameters for these simulated species are summarized in Table 3. The summation of simulated concentration-time profiles of the individual species derived from NKTR-214 agrees with the measured concentration-time profiles of NKTR-214-RC, NKTR-214-AC, and 1-PEG-IL2 (Fig 8). The individual numeric values for the simulated and measured PK parameters (where available) agree within 10% of each other supporting the suitability and initial assumptions of the model (Table 3). It was particularly important to accurately simulate the observed time-course of the most active conjugate, 1-PEG-IL2, which was measured in a separate PK study, further supporting model suitability.

**Table 3:** T_max,_ C_max,_ and AUC of the individual species calculated from the model. Where available, the values from the PK study (Table 2) are shown in parentheses and in the last 2 columns for ease of comparison.

Analyte	T_max_ (hr)	C_max_ (µg/mL)	AUC (µghr/mL)	AUC NKTR-214-RC (µghr/mL)	AUC NKTR-214-AC (µghr/mL)
(4–6)-PEG-IL2	0	11.6	136.6	284.3 (308)	38.3 (37.8)
3-PEG-IL2	14	2.9	109.5		
2-PEG-IL2	25	0.68	31.8		
1-PEG-IL2	28 (24)	0.14 (0.13)	6.4 (5.8)		
IL2	28	0.0006	0.03		

The T_max_ values predicted by the model reflect the PEG release cascade, ranging between 14 hours for 3-PEG-IL2 to 28 hours for 1-PEG-IL2 and theoretical free-IL2. The concentration-time profile of free-IL2 derived from NKTR-214 as predicted by the model is less than 0.08% the AUC of active conjugated-IL2 species derived from NKTR-214. The C_max_ of free-IL2 potentially derived from NKTR-214 is ~20000-fold lower than the C_max_ of NKTR-214 and ~230-fold lower than 1-PEG-IL2, the most active conjugate derived from NKTR-214. The low levels of free-IL2 derived from NKTR-214 simulated by the model are consistent with experimental observations, and support the prediction that free-IL2 is cleared faster than it is released from NKTR-214.

#### Simulation of receptor occupancy for conjugated-IL2 species derived from NKTR-214

**Fig 9:**
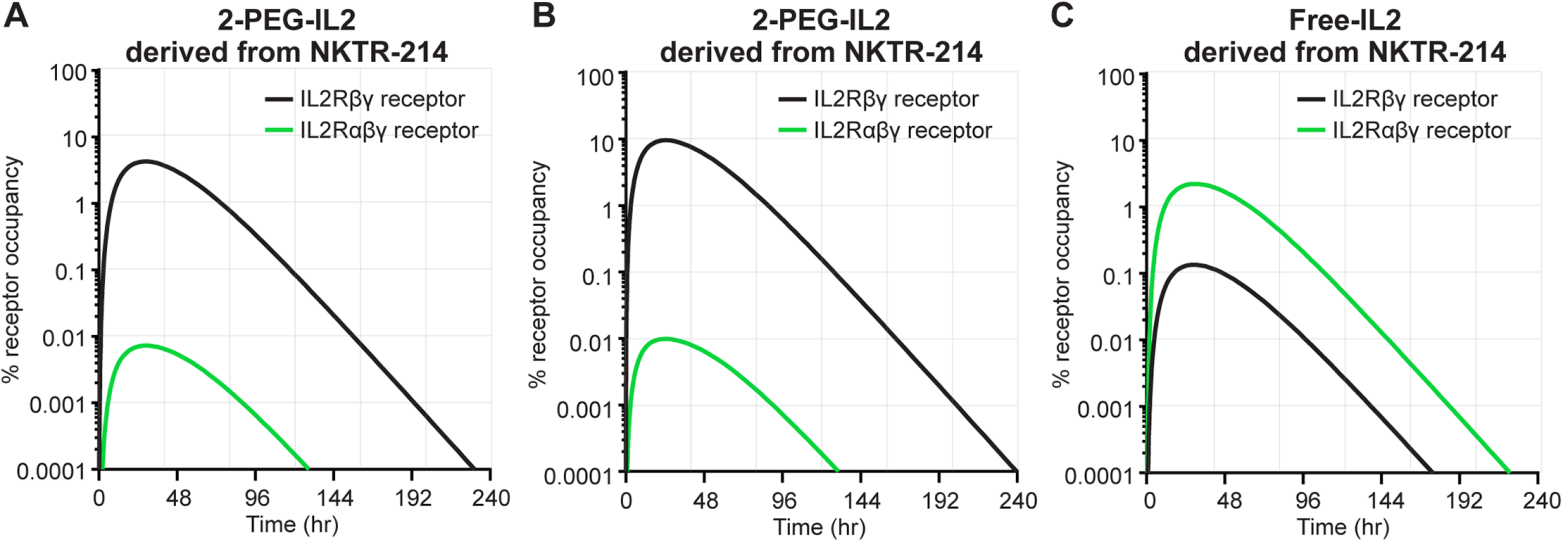
Conjugated-IL2 species derived from NKTR-214 preferentially occupy IL2Rβγ over IL2Rαβγ. Percent receptor occupancy calculated by the model from A. 2-PEG-IL2, B. 1-PEG-IL2, and C. free-IL2 derived from NKTR-214, at IL2Rβγ (gray lines) and IL2Rαβγ (green lines) receptor complexes.

A key goal for developing the mechanistic model was to understand the receptor bias towards the IL2Rβγ over IL2Rαβγ in greater detail. The IL2Rβγ complex is predominantly expressed on CD8 T cells and on NK cells; whereas, IL2Rαβγ is constitutively expressed on Tregs. By unifying all the kinetic data, we could evaluate which receptor complex is predominantly occupied when exposed to the array of conjugated-IL2 species derived from NKTR-214. Although free-IL2 derived from NKTR-214 is undetectable *in vivo* due to its rapid clearance, it was instructive to evaluate the receptor dynamics of the calculated low levels in comparison to the conjugated-IL2 species. Using experimental kinetic parameters and *in vitro* IL2 receptor binding constants, the model indicated that 2-PEG-IL2 and 1-PEG-IL2 are the primary occupants of the IL2Rβγ (Fig 9 and Table 4). Comparing between receptors, after a single dose of NKTR-214, the total occupancy AUC at IL2Rβγ is ~5.9-fold higher than the total occupancy AUC at IL2Rαβγ (695.8 [% receptor occupancy *hr] vs 118.2 [% receptor occupancy*hr], respectively), Table 4. Individually, the 1-PEG-IL2 and 2-PEG-IL2 resulted in ~32-fold and ~70-fold higher IL2Rβγ occupancy compared to free-IL2. These results are reasonable because the affinities of 1-PEG-IL2 and 2-PEG-IL2 for IL2Rβγ are only modestly lower than that of IL2 (7.4- and 16.5-fold less than IL2, respectively) while their AUC of concentration is 233 and 1177 times higher than IL2. In contrast, the affinity of 1-PEG-IL2 or 2-PEG-IL2 for IL2Rαβγ is significantly lower than that of IL2 (3248- and 4668-fold, respectively). Consequently, the 1-PEG-IL2 and 2-PEG-IL2 derived from NKTR-214 only resulted in a fraction of the occupancy by free-IL2 (0.003 and 0.004, respectively) at IL2Rαβγ. This is observable as an inversion of the green and black lines in Fig 9C (IL2) compared to Fig 9A and 9B (NKTR-214). Overall, the model indicates that in the presence of NKTR-214 and its conjugated-IL2 daughter species, IL2Rβγ is the favored receptor complex with an occupancy that is significantly higher than IL2Rαβγ.

**Table 4:** Model-simulated values of receptor occupancy for the conjugated-IL2 species and free-IL2 derived from NKTR-214 after a single dose administration. 1-PEG-IL2 and 2-PEG-IL2 are present at significantly higher levels in plasma than IL2. Experimentally, free-IL2 from NKTR-214 is not detectable. The calculation indicates that individually and in sum, the active conjugated-IL2 from NKTR-214 occupy IL2Rβγ to greater extent than IL2Rαβγ.

IL2-receptor complex	Total AUC of receptor occupancy from NKTR-214[%receptor occupancy*hr]	Calculated AUC receptor occupancy of the most active conjugated species derived from NKTR-214 [%receptor occupancy*hr]		
		2-PEG-IL2 from NKTR-214	1-PEG-IL2 fromNKTR-214	Free-IL2 from NKTR-214
**IL2Rβγ**	695.8	474.6 (68%)	214.4 (31%)	6.8 (1%)
**IL2Rαβγ**	118.2	0.51 (0.4%)	0.38 (0.3%)	117.3 (99.3%)

Values in parenthesis show percentage contribution of each conjugated-IL2 species derived from NKTR-214 relative to total receptor occupancy AUC from all species

### Receptor occupancy of NKTR-214 compared directly to aldesleukin at therapeutic dose levels and exposures

Having established the bias in occupancy of IL2Rβγ by conjugated-IL2 species derived from NKTR-214, we used the model to compare the IL2 receptor occupancy of NKTR-214 to aldesleukin. These comparisons were made to separate the effects of intrinsic receptor bias due to location of the PEG chains, from sustained exposure to active conjugated-IL2, which is also a property provided by the releasable PEG prodrug design. Furthermore, we examined the biased receptor occupancy of NKTR-214 in comparison to the calculated receptor occupancy of a hypothetical “sustained delivery formulation” of aldesleukin.

**Fig 10:**
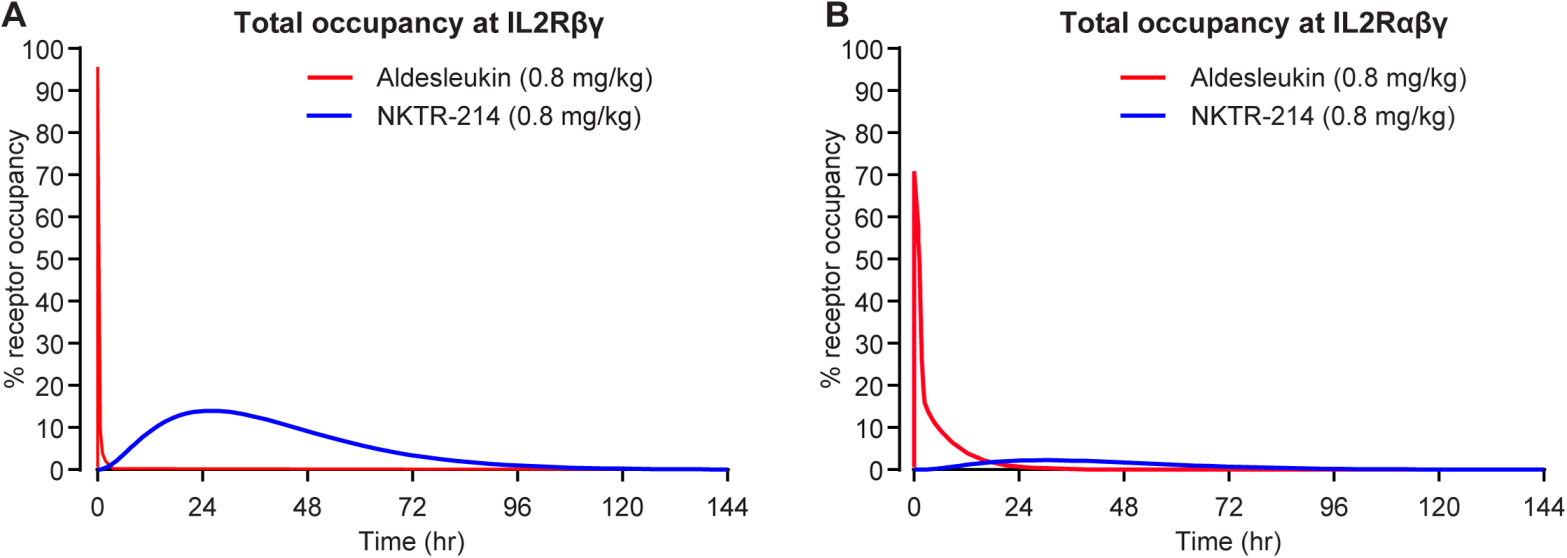
Receptor bias is an intrinsic property of NKTR-214. Simulation of the receptor occupancy percentage over time for therapeutic levels (0.8 mg/kg) of aldesleukin qdx5 (red) or NKTR-214, qd (blue) compared head to head. A. The receptor occupancy area under the curve for IL2Rβγ is 20.8-fold higher after NKTR-214 compared to aldesleukin. B. In contrast, the AUC of IL2Rαβγ is 0.56-fold after NKTR-214 compared to aldesleukin.

Receptor occupancy was first calculated using therapeutic dose levels of NKTR-214 or aldesleukin, each at 0.8 mg/kg. NKTR-214 was provided as a single dose while aldesleukin was administered either as one single dose, 5 daily doses for 2 cycles (a typical efficacious regimen in animal tumor model [16] or a theoretical sustained delivery formulation. The sustained delivery formulation allowed for an aldesleukin AUC that was the same as the AUC for the active conjugated-IL2 species derived from NKTR-214 (NKTR-214-AC). In all 3 scenarios, there was a marked distinction in receptor dynamics between NKTR-214 and aldesleukin administration **(**Table 5). After a single dose of aldesleukin (Fig 10, red line in both panels), the receptor occupancy of IL2Rβγ reached a maximum of 95% immediately after administration but dropped to below 1% in 2 hours, while the receptor occupancy of aldesleukin at IL2Rαβγ reached a maximum of 71% in 35 minutes and dropped to < 1% in about 22 hours. In contrast, NKTR-214 (solid blue line in both panels) gradually increased its receptor occupancy at IL2Rβγ, reaching a maximum of 14% in 25 hours, dropping below 1% after 4 days. At the IL2Rαβγ the occupancy of NKTR-214 reached a maximum of only 2.3% 30 hours after administration and dropped below 1% in 64 hours. Based on the area under the receptor occupancy curve, active conjugated-IL2 species derived from NKTR-214 achieved a 20.8-fold higher occupancy of the IL2Rβγ, and 0.56-fold lower occupancy of the IL2Rαβγ compared to aldesleukin (Fig 10 and Table 5). We next compared multiple dosing of aldesleukin (0.8 mg/kg, qdx5 for 2 cycles) to a single dose of NKTR-214 (0.8 mg/kg). In this analysis, the receptor occupancy AUC of multi-dose aldesleukin at IL2Rβγ reached only half of NKTR-214 (Table 5), while the AUC of receptor occupancy at IL2Rαβγ was 19 times higher than NKTR-214. Finally, to further investigate whether sustained exposure was driving the observed bias achieved by NKTR-214, we simulated a constant infusion of aldesleukin, matching the theoretical AUC of aldesleukin to that actually achieved by NKTR-214-AC after a single dose of NKTR-214. Such an exposure of aldesleukin would not be evaluated experimentally due to the limits of tolerability for such a regimen. In this scenario, a hypothetical continuous aldesleukin administration increased AUC of IL2Rβγ receptor occupancy 3.7-fold higher than that achieved with NKTR-214 (2598.7 vs. 695.8, Table 5) due to the higher intrinsic affinity of IL2 relative to the conjugated-IL2 species. However, aldesleukin AUC of receptor occupancy at IL2Rαβγ concomitantly increased to 172-fold that of NKTR-214 (20336.9 vs. 118.2, Table 5). Therefore the model indicates that NKTR-214 maintains its inherent bias, with a 5.9-fold higher occupancy of IL2Rβγ compared to IL2Rαβγ. On the other hand, both the multi-dose and constant infusion aldesleukin results in 0.16- and 0.13-fold occupancy, respectively, at IL2Rβγ compared to IL2Rαβγ. There is no bias towards IL2Rβγ that is achievable by aldesleukin regardless of dose level, number of doses or continuous exposure. The model reveals that the receptor bias is intrinsic to NKTR-214 by location of the PEG chains at the IL2Rα interface and is not simply a result of improved PK due to PEGylation.

**Table 5:** Aldesleukin does not achieve a bias in receptor occupancy, regardless of dose or exposure. Receptor occupancy of a single dose of NKTR-214 compared to several dosing regimens of aldesleukin, including a theoretical sustained release formulation that provides the same measured AUC as NKTR-214. The values are normalized to the AUC of aldesleukin single dose for each receptor complex.

	AUC of receptor occupancy for aldesleukin at 3 dosing regimens compared to single dose NKTR-214 at IL2Rαβγ and IL2Rβγ [%receptor occupancy*hr]			
Receptor	Aldesleukin, 0.8 mg/kg, single dose	Aldesleukin, 0.8 mg/kg, qdx5, 2 cycles^1^	Aldesleukin, 0.8 mg/kg at equivalent AUC to NKTR-214^2^	NKTR-214, 0.8 mg/kg, single dose
**IL2Rβγ**	33.4	357.4	2598.7	695.8
**IL2Rαβγ**	212.6	2265.8	20336.9	118.2
**Ratio of IL2Rβγ to IL2Rαβγ**	**0.16**	**0.16**	**0.13**	**5.9**

^1^Therapeutic aldesleukin regimen typically used in animal tumor models

^2^Theoretical “sustained delivery formulation” of aldesleukin that provides an AUC comparable to NKTR-214. The aldesleukin concentration was artificially kept constant, such that the AUC_0-10d_ of IL2 was the same as the actual AUC_0-10d_ of NKTR-214-AC after a single 0.8 mg/kg dose of NKTR-214.

### Simulation of %pSTAT5 downstream of receptor occupancy

**Fig 11:**
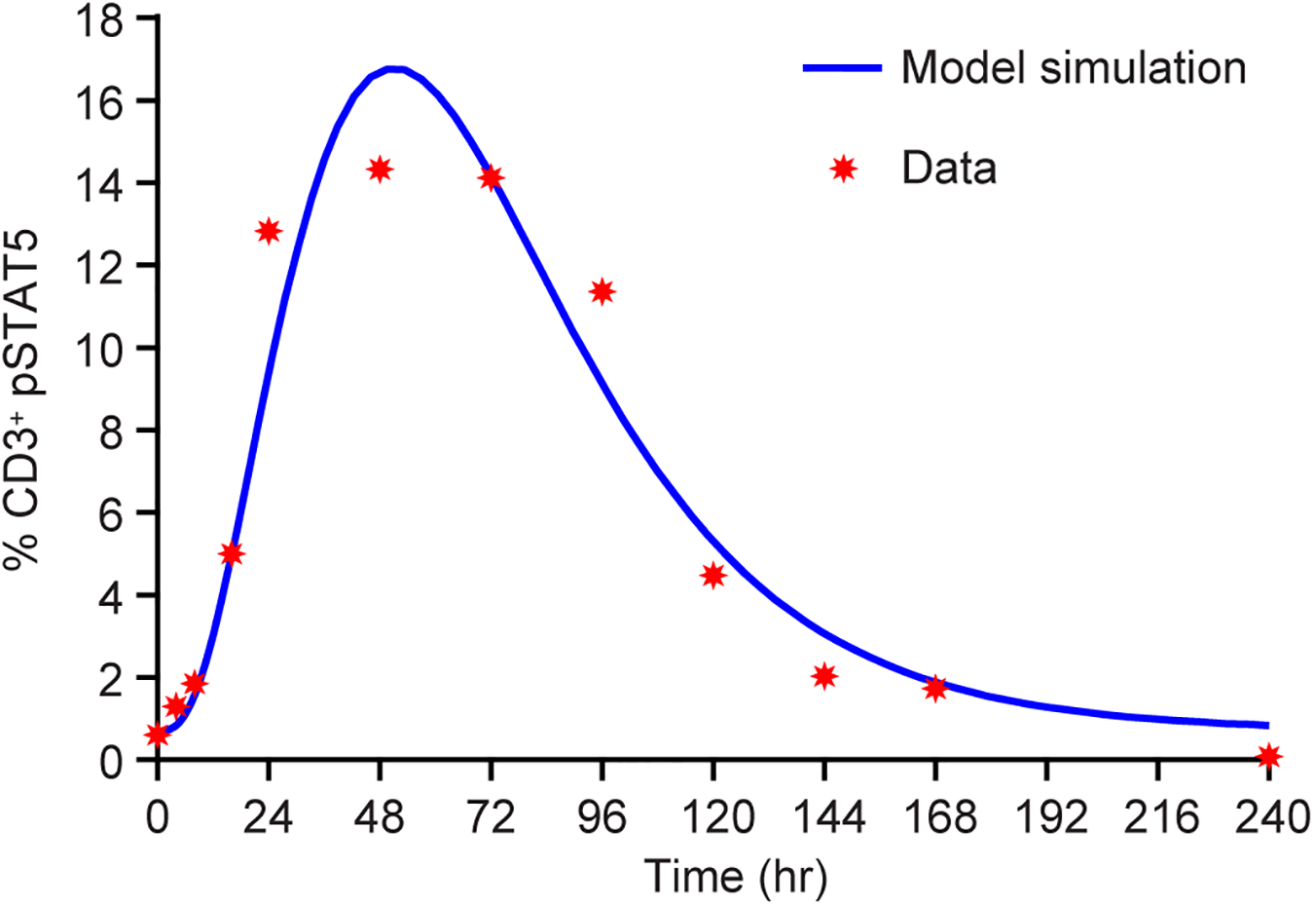
The model correctly estimates experimental % pSTAT5 activation from the receptor occupancy determination. Model fit (blue line) of pSTAT5 signaling from NKTR-214 as compared to the observed measured values (symbols).

A simulation of pSTAT5 activation of T cells as a result of receptor occupancy, as discussed in the Methods section, predicts the experimentally measured pSTAT5 pharmacodynamic data for NKTR-214 (Fig 11). The simulated T_max_ of pSTAT5 is ~50 hours, about one day later than T_max_ of NKTR-214-AC. The data are suggestive of the potential ability of the model to predict downstream biology and pharmacodynamics.

### Impact of NKTR-214 design on *in vivo* antitumor efficacy

**Fig 12:**
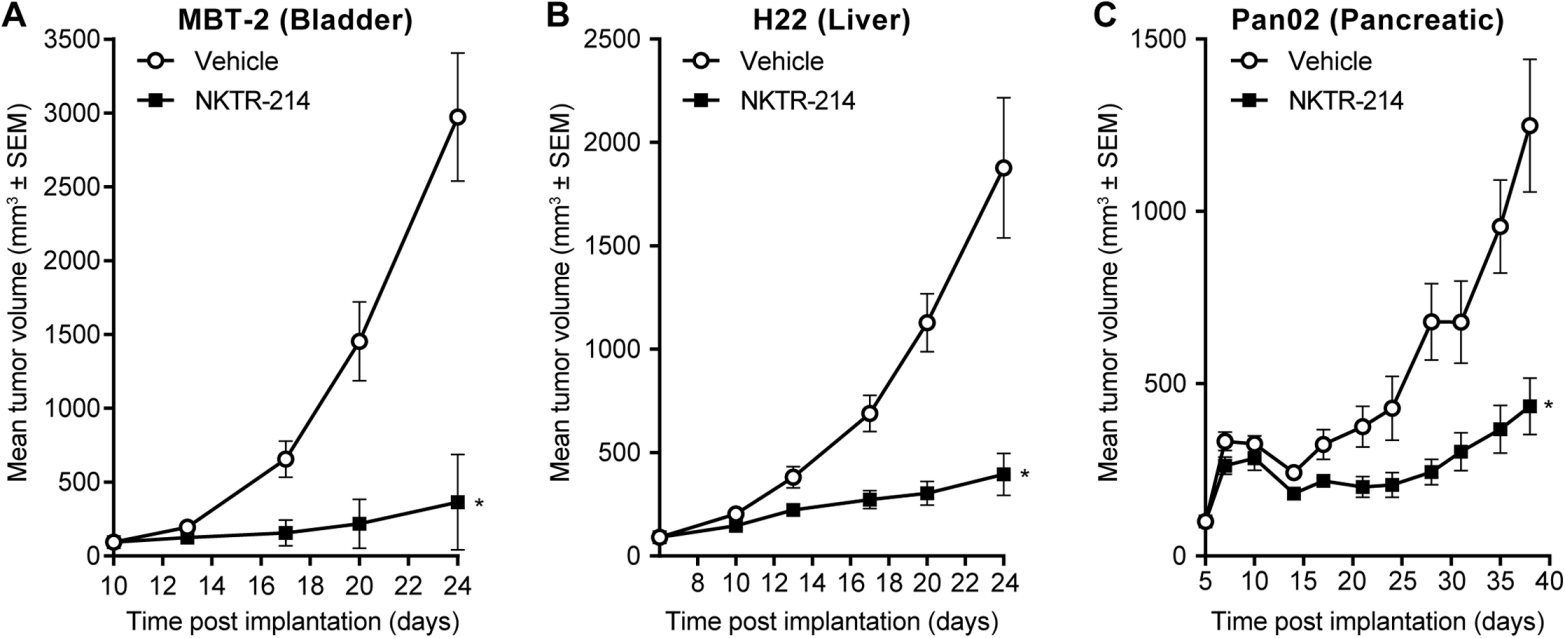
NKTR-214 is consistently effective across several established mouse tumor models. Examples shown below include A. bladder (MBT-2), B. liver (H22), and C. pancreatic (Pan02) for single-agent NKTR-214 compared to vehicle. In all cases, tumors were grown to large size, 80-100mm^3^, N = 7/group; NKTR-214 0.8 mg/kg q9dx3.*, *P* < 0.05 (unpaired t-test).

NKTR-214 was designed with a receptor bias to increase NK and CD8 T cells in the tumor over Tregs to improve anti-tumor efficacy and systemic tolerability. As a single agent in the mouse B16F10 melanoma model and in combination with anti-cytotoxic T-lymphocyte associated protein 4 (CTLA-4) in the EMT-6 mouse breast cancer model, NKTR-214 delivered significantly greater efficacy and improved tolerability than aldesleukin [16]. Importantly, a marked increase in the ratio of CD8/Tregs in the tumor was obtained after one single dose of NKTR-214 (>400) compared to ~18 for five daily doses of aldesleukin. The magnitude of the cellular changes in the tumor microenvironment suggested that the receptor bias combined with sustained tumor exposure served to markedly tip the immunological balance specifically in the tumor as the CD8/Treg was only ~10 in peripheral tissues. Having here quantified and validated the magnitude of the receptor bias from the mechanistic modeling, it became clear how these altered receptor dynamics could potentially translate into greater anti-tumor efficacy. We therefore sought to pressure-test the generality and depth of the anti-tumor response in multiple murine models as a single agent. A panel of syngeneic mouse tumor models were chosen with the aim of testing efficacy against large established tumors (~100 mm^3^) having a well-characterized range of resident tumor infiltrating lymphocytes (TILs) prior to treatment [34, 35]. Consistently, NKTR-214 as a single-agent demonstrated significant tumor growth inhibition (TGI) across all three tumor types tested: bladder (MBT-2, 92%), liver (H22, 79%), and pancreatic (Pan02, 65%) carcinoma (Fig 12).

## Discussion

NKTR-214 has a multi-component kinetically-driven mechanism of action that includes: 1) a pro-drug design that renders NKTR-214 inactive at the time of administration; 2) releasable PEG chains that over time progressively provide conjugated-IL2 species of greater potency; 3) biased receptor binding such that PEGylation impacts binding at IL-2Rαβγ to a greater extent than IL-2Rβγ compared to IL2; and 4) long t_½_ enabling antibody-like dosing regimen in humans. In addition to the important benefit of an improved dosing regimen provided by PEGylation, NKTR-214 is designed with a time-dependent biological activity ranging from inactive in its fully-PEGylated form to active when only 1 and 2 PEG chains remain. At the same time, each of the generated species in the cascade provides biased receptor dynamics that favor interaction with IL2Rβγ, with ultimate activation of CD8 and NK cells in the tumor.

We took a reductionist approach to characterize the NKTR-214 mechanism of action and measured reaction rates for PEG release, receptor association and dissociation, *in vivo* PK, and *in vivo* PD of IL2 pathway engagement. We then assembled these components into a mechanism-based mathematical model to simplify the description of NKTR-214. This model adequately captured our observed results and described the differentiation of NKTR-214 relative to aldesleukin not only with respect to PK, but also with respect to the receptor-binding dynamics that ultimately lead to significant immunological effects in the tumor microenvironment.

Free-IL2 from NKTR-214 is undetectable *in vivo* due to its fast clearance and slow rate of formation. The results from the model support this finding as after administration of NKTR-214, the theoretcally calculated C_max_ of free-IL2 derived from NKTR-214 is ~20000-fold lower than that of PEG-conjugates derived from NKTR-214. It was instructive to understand how conjugated-IL2 species derived from NKTR-214 dominate the activation of IL2Rβγ through greater and prolonged occupancy of this receptor over IL2Rαβγ. The motivation for understanding this phenomenon stems from our previous observation that NKTR-214 significantly mobilizes and increases the number of CD8 T cells in the tumor microenvironment, while Treg numbers remain unchanged or decline [16]. This biological effect is now explained by the percent receptor occupancy shown in Fig 9. While adding PEG reduces the affinity to all IL2 receptors, the affinity of 1-PEG-IL2 and 2-PEG-IL2 at IL2Rβγ is only modestly lower than that of free-IL2 (7.4- and 16.5-fold, respectively) due to the distal location of the PEG chains. Given their exposures are much higher than IL2, these NKTR-214 conjugates successfully form receptor complexes with IL2Rβγ. In contrast, the affinity of 1-PEG-IL2 and 2-PEG-IL2 at IL2Rαβγ is significantly lower than that of free-IL2 (3247-fold and 4669-fold lower, respectively); even with the greater exposure they still only reach a fraction of the occupancy of free-IL2 at this receptor. Consequently, the conjugated-IL2 species occupy the IL2Rβγ more readily than IL2Rαβγ and this property is an inherent feature of NKTR-214 that promotes its favorable biological and therapeutic effects in murine tumor models (Fig 12) [16]. A theoretical “sustained delivery formulation” of aldesleukin with an AUC matched to the AUC of NKTR-214 is unable to achieve the receptor bias of NKTR-214 (Table 5), indicating that IL2 exposure alone is unable to account for the receptor bias. Overall, the data and mechanistic modeling suggest that the marked anti-tumor activity of NKTR-214 arises from the combination of sustained exposure and receptor bias, both of which are built into the molecular design of the agent.

While the model accurately fits the PK and PD data for NKTR-214, several assumptions were made as discussed in the Methods section. For example, more information is needed to fully understand the *in vivo* release rates, tissue distribution, and clearance of each conjugated-IL2 species to gain greater insight into the pharmacokinetics. A thorough assessment of the pharmacodynamics, in particular pSTAT5 signaling in indivudal T cell populations will provide a comprehensive understanding of the immunological dynamics. Given that NKTR-214 is a unique biologic drug that evolves with time, we expect the model to evolve as these data become available through development of additional bioanalytical assays and new *in vivo* mechanistic studies.

The receptor biased component of NKTR-214's kinetically-controlled mechanism of action is significantly different from previously described controlled-release formulations, long-acting IL2 formulations, Fc fusions, or antibody-IL2 complexes. For example, IL2 has previously been formulated to allow slow release from a polymer matrix [30, 31, 36]. -IL2 release from such a matrix does not create an array of conjugated-IL2 species such as that produced from NKTR-214 *in vivo*. As described, matching the theoretical exposure of IL2 to that of NKTR-214 does not result in the biased receptor occupancy achieved by NKTR-214 [32, 33]. Moreover, these IL2 formulations, Fc fusions, or antibody complexes immediately deliver active IL2 to the system, potentially increasing toxicities. In contrast, active conjugated-IL2 species derived from NKTR-214 are provided in a gradual kinetically-controlled cascade over time. In another approach, screening technologies have identified IL2 muteins that demonstrate exquisite affinity towards IL2Rβγ that are several hundred-fold higher than IL2Rαβγ [37]. These IL2Rβγ-selective muteins contrast sharply with the subtle bias introduced by the design of NKTR-214 which does not completely eliminate affinity to IL2Rαβγ once activated. This is important because many immune cells transiently up-regulate IL2Rαβγ upon activation to increase IL2 sensitivity when mounting an immunological response, including priming of CD8 T cells [38]. Therefore, some IL2Rαβγ binding is necessary and this is provided by NKTR-214. Using PEGylation as a means of achieving receptor bias is a novel use of PEGylation technology. As a polymeric material, PEG is generally regarded as safe, and its long history in many FDA approved products may offer a measure of confidence [39].

The receptor biased component of the NKTR-214 mechanism of action likely relates to the anti-tumor efficacy of the molecule by focusing its activity on CD8 and NK cells in the tumor microenvironment. At the same time, the prodrug design of NKTR-214 mitigates immune system over-activation by providing sustained and controlled IL2 pathway signaling as measured by pSTAT5. A key issue of high-dose IL2 (HD-IL2) therapy is the multiple rapid-activation peaks that patients must endure once every 8 hours. This regimen leads to side effects including severe hypotension, neurotoxicity, cytokine storm, and vascular leakage. With NKTR-214, the multiple peaks intrinsic to HD-IL2 are absent. As anticipated, NKTR-214 has shown good tolerability in rat and non-human primate preclinical toxicology studies, enabling initiation of ongoing clinical studies. Although speculative, it is conceivable that the residual IL2Rαβγ binding allowed by NKTR-214 activates protective Tregs in the peripheral blood where the CD8/Treg ratio is ~40-fold lower than that observed in the tumor [16]. This effect is consistent with activation of Tregs by low-dose IL2 therapy [40] perhaps engendered by the low level of IL2Rαβγ occupancy as described by the model herein. The kinetically-controlled pharmacology arising from NKTR-214 provides a means of achieving marked efficacy and tolerability in multiple murine tumors as shown.

NKTR-214, as both a monotherapy and in combination with anti-PD-1 is currently in Phase 1 / 2 trials for the treatment of solid tumors in an outpatient setting. Given the requirement of the IL2 pathway for T cell activation and proliferation, NKTR-214 may serve as a backbone therapy that enhances multiple anti-tumor modalities. Finally, although NKTR-214 is a cytokine-based therapy, it is dosed like an antibody in human studies, administered once every 2 weeks or once every 3 weeks in the clinic. This regimen is particularly beneficial for patients also receiving checkpoint inhibitor antibodies as these agents are similarly dosed once every 2 or 3 weeks.

Cytokines represent a potent endogenous class of molecules with clear ability to robustly modulate the immune system. Turning these molecules into effective and practical medicines will be an important component of pushing the immuno-oncology revolution to the next level so more patients might achieve a durable response or perhaps even a cure.

## References

[1] McDermott DF, Sosman JA, Sznol M, Massard C, Gordon MS, Hamid O, et al Atezolizumab, an Anti-Programmed Death-Ligand 1 Antibody, in Metastatic Renal Cell Carcinoma: Long-Term Safety, Clinical Activity, and Immune Correlates From a Phase Ia Study. J Clin Oncol. 2016;34(8):833–42. doi: 10.1200/JCO.2015.63.7421 . 26755520 10.1200/JCO.2015.63.7421

[2] Sznol M, Longo DL. Release the Hounds! Activating the T-Cell Response to Cancer. The New England journal of medicine. 2015;372(4):374–5. doi: 10.1056/NEJMe1413488 . 25482238 10.1056/NEJMe1413488

[3] McDermott DF, Drake CG, Sznol M, Choueiri TK, Powderly JD, Smith DC, et al Survival, Durable Response, and Long-Term Safety in Patients With Previously Treated Advanced Renal Cell Carcinoma Receiving Nivolumab. J Clin Oncol. 2015;33(18):2013–20. doi: 10.1200/JCO.2014.58.1041 ; PubMed Central PMCID: PMCPMC4517051. 25800770 10.1200/JCO.2014.58.1041 PMC4517051

[4] Assal A, Kaner J, Pendurti G, Zang X. Emerging targets in cancer immunotherapy: beyond CTLA-4 and PD-1. Immunotherapy. 2015;7(11):1169–86. doi: 10.2217/imt.15.78 . 26567614 10.2217/imt.15.78 PMC4976877

[5] Le Mercier I, Lines JL, Noelle RJ. Beyond CTLA-4 and PD-1, the Generation Z of Negative Checkpoint Regulators. Frontiers in immunology. 2015;6:418 doi: 10.3389/fimmu.2015.00418 ; PubMed Central PMCID: PMCPMC4544156. 26347741 10.3389/fimmu.2015.00418 PMC4544156

[6] Luheshi NM, Coates-Ulrichsen J, Harper J, Mullins S, Sulikowski MG, Martin P, et al Transformation of the tumour microenvironment by a CD40 agonist antibody correlates with improved responses to PD-L1 blockade in a mouse orthotopic pancreatic tumour model. Oncotarget. 2016;7(14):18508–20. doi: 10.18632/oncotarget.7610 ; PubMed Central PMCID: PMCPMC4951305. 26918344 10.18632/oncotarget.7610 PMC4951305

[7] Lipiainen T, Peltoniemi M, Sarkhel S, Yrjonen T, Vuorela H, Urtti A, et al Formulation and stability of cytokine therapeutics. Journal of pharmaceutical sciences. 2015;104(2):307–26. doi: 10.1002/jps.24243 . 25492409 10.1002/jps.24243

[8] Schein CH. The shape of the messenger: using protein structure information to design novel cytokine-based therapeutics. Curr Pharm Des. 2002;8(24):2113–29. . 12369857 10.2174/1381612023393161 PMC11963425

[9] Sim GC, Radvanyi L. The IL-2 Cytokine Family in Cancer Immunotherapy. Cytokine & Growth Factor Reviews. 2014 doi: 10.1016/j.cytogfr.2014.07.018 25200249 10.1016/j.cytogfr.2014.07.018

[10] McDermott DF, Regan MM, Clark JI, Flaherty LE, Weiss GR, Logan TF, et al Randomized phase III trial of high-dose interleukin-2 versus subcutaneous interleukin-2 and interferon in patients with metastatic renal cell carcinoma. J Clin Oncol. 2005;23(1):133–41. doi: 10.1200/JCO.2005.03.206 . 15625368 10.1200/JCO.2005.03.206

[11] Payne R, Glenn L, Hoen H, Richards B, Smith JW 2nd, Lufkin R, et al Durable responses and reversible toxicity of high-dose interleukin-2 treatment of melanoma and renal cancer in a Community Hospital Biotherapy Program. J Immunother Cancer. 2014;2:13 Epub 2014/05/24. doi: 10.1186/2051-1426-2-13 ; PubMed Central PMCID: PMCPmc4030280. 24855563 10.1186/2051-1426-2-13 PMC4030280

[12] Alexandrescu DT, Maddukuri P, Wiernik PH, Dutcher JP. Thrombotic thrombocytopenic purpura/hemolytic uremic syndrome associated with high-dose interleukin-2 for the treatment of metastatic melanoma. J Immunother. 2005;28(2):144–7. . 15725958 10.1097/01.cji.0000154250.82007.4a

[13] Boyman O, Sprent J. The role of interleukin-2 during homeostasis and activation of the immune system. Nature reviews Immunology. 2012;12(3):180–90. doi: 10.1038/nri3156 . 22343569 10.1038/nri3156

[14] Dutcher J. Current status of interleukin-2 therapy for metastatic renal cell carcinoma and metastatic melanoma. Oncology (Williston Park). 2002;16(11 Suppl 13):4–10. . 12469934

[15] Letourneau S, van Leeuwen EM, Krieg C, Martin C, Pantaleo G, Sprent J, et al IL-2/anti-IL-2 antibody complexes show strong biological activity by avoiding interaction with IL-2 receptor alpha subunit CD25. Proc Natl Acad Sci U S A. 2010;107(5):2171–6. doi: 10.1073/pnas.0909384107 ; PubMed Central PMCID: PMC2836659. 20133862 10.1073/pnas.0909384107 PMC2836659

[16] Charych DH, Hoch U, Langowski JL, Lee SR, Addepalli MK, Kirk PB, et al NKTR-214, an Engineered Cytokine with Biased IL2 Receptor Binding, Increased Tumor Exposure, and Marked Efficacy in Mouse Tumor Models. Clin Cancer Res. 2016;22(3):680–90. doi: 10.1158/1078-0432.CCR-15-1631 . 26832745 10.1158/1078-0432.CCR-15-1631

[17] https://www.drugbank.ca/drugs/DB00041. Aldesleukin. DrugBank. (DB00041 (BTD00082, BIOD00082)):DB00041 (BTD82, BIOD82). DB00041 (BTD00082, BIOD00082).

[18] Leon K, Garcia-Martinez K, Carmenate T. Mathematical Models of the Impact of IL2 Modulation Therapies on T Cell Dynamics. Frontiers in immunology. 2013;4:439 doi: 10.3389/fimmu.2013.00439 ; PubMed Central PMCID: PMCPMC3858650. 24376444 10.3389/fimmu.2013.00439 PMC3858650

[19] Busse D, de la Rosa M, Hobiger K, Thurley K, Flossdorf M, Scheffold A, et al Competing feedback loops shape IL-2 signaling between helper and regulatory T lymphocytes in cellular microenvironments. Proceedings of the National Academy of Sciences of the United States of America. 2010;107(7):3058–63. doi: 10.1073/pnas.0812851107 ; PubMed Central PMCID: PMCPMC2840293. 20133667 10.1073/pnas.0812851107 PMC2840293

[20] Feinerman O, Jentsch G, Tkach KE, Coward JW, Hathorn MM, Sneddon MW, et al Single-cell quantification of IL-2 response by effector and regulatory T cells reveals critical plasticity in immune response. Mol Syst Biol. 2010;6:437 doi: 10.1038/msb.2010.90 ; PubMed Central PMCID: PMCPMC3010113. 21119631 10.1038/msb.2010.90 PMC3010113

[21] Garcia-Martinez K, Leon K. Modeling the role of IL2 in the interplay between CD4+ helper and regulatory T cells: studying the impact of IL2 modulation therapies. International immunology. 2012;24(7):427–46. doi: 10.1093/intimm/dxr120 . 22371423 10.1093/intimm/dxr120

[22] Shanafelt AB, Lin Y, Shanafelt MC, Forte CP, Dubois-Stringfellow N, Carter C, et al A T-cell-selective interleukin 2 mutein exhibits potent antitumor activity and is well tolerated in vivo. Nature biotechnology. 2000;18(11):1197–202. doi: 10.1038/81199 . 11062441 10.1038/81199

[23] Taniguchi T, Minami Y. The IL-2/IL-2 receptor system: a current overview. Cell. 1993;73(1):5–8. . 8462103 10.1016/0092-8674(93)90152-g

[24] Stauber DJ, Debler EW, Horton PA, Smith KA, Wilson IA. Crystal structure of the IL-2 signaling complex: paradigm for a heterotrimeric cytokine receptor. Proc Natl Acad Sci U S A. 2006;103(8):2788–93. doi: 10.1073/pnas.0511161103 ; PubMed Central PMCID: PMCPMC1413841. 16477002 10.1073/pnas.0511161103 PMC1413841

[25] Wang X, Rickert M, Garcia KC. Structure of the quaternary complex of interleukin-2 with its alpha, beta, and gammac receptors. Science. 2005;310(5751):1159–63. doi: 10.1126/science.1117893 . 16293754 10.1126/science.1117893

[26] Wu Z, Johnson KW, Choi Y, Ciardelli TL. Ligand binding analysis of soluble interleukin-2 receptor complexes by surface plasmon resonance. The Journal of biological chemistry. 1995;270(27):16045–51. . 7608166 10.1074/jbc.270.27.16045

[27] Myszka DG, Arulanantham PR, Sana T, Wu Z, Morton TA, Ciardelli TL. Kinetic analysis of ligand binding to interleukin-2 receptor complexes created on an optical biosensor surface. Protein science: a publication of the Protein Society. 1996;5(12):2468–78. doi: 10.1002/pro.5560051209 ; PubMed Central PMCID: PMC2143301. 8976555 10.1002/pro.5560051209 PMC2143301

[28] Stauber DJ, Debler EW, Horton PA, Smith KA, Wilson IA. Crystal structure of the IL-2 signaling complex: Paradigm for a heterotrimeric cytokine receptor. Proceedings of the National Academy of Sciences of the United States of America. 2006;103(8):2788–93. doi: 10.1073/pnas.0511161103 16477002 10.1073/pnas.0511161103 PMC1413841

[29] Rickert M, Boulanger MJ, Goriatcheva N, Garcia KC. Compensatory energetic mechanisms mediating the assembly of signaling complexes between interleukin-2 and its alpha, beta, and gamma(c) receptors. J Mol Biol. 2004;339(5):1115–28. doi: 10.1016/j.jmb.2004.04.038 . 15178252 10.1016/j.jmb.2004.04.038

[30] Konigsberg PJ, Godtel R, Kissel T, Richer LL. The development of IL-2 conjugated liposomes for therapeutic purposes. Biochimica et biophysica acta. 1998;1370(2):243–51. . 9545572 10.1016/s0005-2736(97)00269-1

[31] Bos GW, Jacobs JJ, Koten JW, Van Tomme S, Veldhuis T, van Nostrum CF, et al In situ crosslinked biodegradable hydrogels loaded with IL-2 are effective tools for local IL-2 therapy. Eur J Pharm Sci. 2004;21(4):561–7. doi: 10.1016/j.ejps.2003.12.007 . 14998588 10.1016/j.ejps.2003.12.007

[32] Craiu A, Barouch DH, Zheng XX, Kuroda MJ, Schmitz JE, Lifton MA, et al An IL-2/Ig fusion protein influences CD4+ T lymphocytes in naive and simian immunodeficiency virus-infected Rhesus monkeys. AIDS Res Hum Retroviruses. 2001;17(10):873–86. doi: 10.1089/088922201750290005 . 11461674 10.1089/088922201750290005

[33] Penichet ML, Harvill ET, Morrison SL. Antibody-IL-2 fusion proteins: a novel strategy for immune protection. Hum Antibodies. 1997;8(3):106–18. . 9322080

[34] Zhang L, Zhang J, Shi Q, editors. Abstract A6: RNAseq and immune profiling analysis of syngeneic mouse models treated with immune checkpoint inhibitors enable biomarker discovery and model selection for cancer immunotherapy. Molecular Cancer Therapeutics; 2015.

[35] Zhang L, Zhang J, Shi Q. Abstract A6: RNAseq and immune profiling analysis of syngeneic mouse models treated with immune checkpoint inhibitors enable biomarker discovery and model selection for cancer immunotherapy. Molecular Cancer Therapeutics. 2015;14(12 Supplement 2):A6–A. doi: 10.1158/1535-7163.targ-15-a6

[36] Samlowski WE, McGregor JR, Jurek M, Baudys M, Zentner GM, Fowers KD. ReGels Polymer-based Delivery of Interleukin-2 as a Cancer Treatment. Journal of immunotherapy. 2006;29(5):524–35. doi: 10.1097/01.cji.0000211306.05869.25 16971808 10.1097/01.cji.0000211306.05869.25

[37] Levin AM, Bates DL, Ring AM, Krieg C, Lin JT, Su L, et al Exploiting a natural conformational switch to engineer an interleukin-2 'superkine'. Nature. 2012;484(7395):529–33. doi: 10.1038/nature10975 ; PubMed Central PMCID: PMC3338870. 22446627 10.1038/nature10975 PMC3338870

[38] Kalia V, Sarkar S, Subramaniam S, Haining WN, Smith KA, Ahmed R. Prolonged interleukin-2Ralpha expression on virus-specific CD8+ T cells favors terminal-effector differentiation in vivo. Immunity. 2010;32(1):91–103. doi: 10.1016/j.immuni.2009.11.010 . 20096608 10.1016/j.immuni.2009.11.010

[39] Fishburn CS. The pharmacology of PEGylation: balancing PD with PK to generate novel therapeutics. Journal of pharmaceutical sciences. 2008;97(10):4167–83. doi: 10.1002/jps.21278 . 18200508 10.1002/jps.21278

[40] Koreth J, Matsuoka K, Kim HT, McDonough SM, Bindra B, Alyea EP 3rd, et al Interleukin-2 and regulatory T cells in graft-versus-host disease. The New England journal of medicine. 2011;365(22):2055–66. doi: 10.1056/NEJMoa1108188 ; PubMed Central PMCID: PMC3727432. 22129252 10.1056/NEJMoa1108188 PMC3727432

